# Maternal short chain fructo-oligosaccharides supplementation during late gestation and lactation influences milk components and offspring gut metabolome: a pilot study

**DOI:** 10.1038/s41598-024-54813-3

**Published:** 2024-02-20

**Authors:** Cindy Le Bourgot, Virginie Lollier, Yoann Richer, Loric Thoulouze, Ljubica Svilar, Sophie Le Gall, Sophie Blat, Isabelle Le Huërou-Luron

**Affiliations:** 1 Tereos, Scientific and Regulatory Affairs Department, Moussy-le-Vieux, France; 2 INRAE, UR1268 BIA, 44300 Nantes, France; 3 INRAE, PROBE Research Infrastructure, BIBS Facility, 44300 Nantes, France; 4 Cribiom, Centre de Recherche Cardiovasculaire et Nutrition C2VN, UMR INRAE 1260 INSERM 1263, University Aix-Marseille, Marseille, France; 5 Institut NuMeCan, INRAE, INSERM, University Rennes, 35590 Saint-Gilles, France

**Keywords:** Prebiotic, Maternal nutrition, Gut microbiota, Milk oligosaccharides, Milk lipidome, Faecal metabolome, Physiology, Nutrition, Paediatrics

## Abstract

Breast milk composition is influenced by maternal diet. This study aimed to evaluate if supplementation of maternal diet with a prebiotic fibre, through its potential effect on milk composition, can be a leverage to orientate the gut microbiota of infants in a way that would be beneficial for their health. Twelve sows received a diet supplemented with short chain fructo-oligosaccharides or maltodextrins during the last month of gestation and the lactation. Oligosaccharidic and lipidomic profiles of colostrum and mature milk (21 days), as well as faecal microbiota composition and metabolomic profile of 21 day-old piglets were evaluated. The total porcine milk oligosaccharide concentration tended to be lower in scFOS-supplemented sows, mainly due to the significant reduction of the neutral core oligosaccharides (in particular that of a tetrahexose). Maternal scFOS supplementation affected the concentration of 31 lipids (mainly long-chain triglycerides) in mature milk. Faecal short-chain fatty acid content and that of 16 bacterial metabolites were modified by scFOS supplementation. Interestingly, the integrative data analysis gave a novel insight into the relationships between (i) maternal milk lipids and PMOs and (ii) offspring faecal bacteria and metabolites. In conclusion, scFOS-enriched maternal diet affected the composition of mature milk, and this was associated with a change in the colonisation of the offspring intestinal microbiota.

## Introduction

Several studies have reported the maternal contribution to infant health, including the effect of maternal diet during pregnancy and lactation. Maternal diet may impact gut microbiota development of infants and subsequently, their health outcomes, through the modulation of maternal bacteria transferred vertically to the newborn at delivery and the evolution of breast milk composition during lactation^1–3^.

Breast milk is the optimal source of nutrition for the first few months of infant life, as it provides all the necessary nutrients for optimal growth and development. Breast milk is composed of lactose and lipids, the two main sources of energy, and of proteins and oligosaccharides^1,4^. Milk oligosaccharides (MOs) act as bioactive components conferring benefits to the early development of infant gut microbiota^5^. MOs are typically composed of 3 to 10 monosaccharide units, including glucose (Glc), galactose (Gal), *N*-acetyl-glucosamine (GlcNAc), *N*-acetyl-galactosamine (GalNAc), fucose (Fuc), and sialic acids (NeuAc). Based on their chemical composition, MOs are classified as acidic (decorated by sialic acid, NeuAc), neutral core, or fucosylated MOs. Due to their structure, MOs are not hydrolysed by digestive enzymes and reach the distal intestine where they directly influence the gut microbiota composition in breastfed infants^6^. Indeed, they are used as energy source by some specific bacteria, such as *Bifidobacterium*, for their growth, and they act as antimicrobial and antiadhesive agents^7,8^. They can also act indirectly by modulating infant immune response^8^. In addition to MOs, milk lactose content and, to a lesser extent, that of proteins and lipids were reported to influence faecal microbiota composition in piglets^9^. The structure and composition of triglycerides, fatty acids, phospholipids, and sphingolipids may differently impact the primocolonization of the gut^10–15^. As an example, an infant formula supplementation with milk fat globule membrane (MFGM) has been shown to shift the faecal microbiota of supplemented-infant formula fed neonatal piglets towards the composition of sow-reared piglets^16^. Similar results were observed in rats^17^. Finally, the impact of proteins on microbiota shaping is frequently associated to protein having immunomodulatory properties such as lactoferrin, lysozyme, immunoglobulins, but the nature of whey proteins may also play a role in infant formulas^1,18^.

The use of prebiotic fibres, including short chain fructo-oligosaccharides (scFOS), as dietary supplements, is a nutritional strategy recognized for modulating the intestinal microbiota. Our group previously demonstrated that maternal scFOS supplementation during the last 4 weeks of gestation and the whole lactation in sows modulated the offspring gut microbiota composition with beneficial consequences on the host physiology in infant piglets and later on, in adulthood^19,20^. However, the contribution of sow milk composition to the observed changes of offspring microbiota composition was not studied, although an increase in lipid content and energy value, and a tendency for more proteins in milk of sows supplemented with scFOS were observed.

The effect of maternal diet on milk macronutrients mainly implies the qualitative effects of maternal diet lipid profile on that of its milk^21,22^. In contrast, the effect of the nutritional profile of the maternal diet in terms of protein, fat or fibre on MO composition has so far been little investigated^1^. A recent scoping review including fourteen studies identified potential associations between maternal dietary intake and the MOs^23^. For instance, maternal fruit consumption, a dietary source of simple sugar and dietary fibres, was positively correlated with absolute amounts of selected MOs and with levels of Gal and Fuc present in these MOs, while NeuAc levels were lower^24^. Similarly, Azad et al. (2018) found a positive correlation between wholegrains in the maternal diet and a fucosylated oligosaccharide (fucosyllacto-*N*-hexaose) in their milk^25^. A recent study demonstrated that distinct maternal dietary carbohydrate (Glc *vs* Gal) and energy (carbohydrates *vs* high fat) sources alter MO composition, including fucosylated species^26^ whereas lipid-based nutrient supplements during pregnancy and lactation did not affect human MOs (HMOs)^27^. Finally, a study investigating the consequences of probiotic supplements given during the late stage of pregnancy demonstrated a change in the relative MO composition and profile, with an increase in 3’-fucosyllactose (3’-FL) and 3'-sialyllactose (3’-SL) while the total amount of MOs decreased^28^. Overall, these studies revealed an impact that seems to be specific for each class of MOs. However, the impact of maternal diet supplementation with a prebiotic fibre on MO composition and lipidome has never been explored.

Only few studies have evaluated porcine MO (PMO) changes during early lactation^29–33^. Piglets are considered a suitable model for human infant nutrition due to similarities in the development of intestinal physiology and nutrient requirements^34^. In addition, while major animal MOs are sialylated and fucosylated, PMOs have been reported to be more similar to HMOs than bovine MOs^30,31^. Indeed, human milk and porcine milk share about ten oligosaccharides, including the PMO predominant acidic (3’-SL, Neu5Ac(α2–6)Gal(β1–4)GlcNAc and 6’-SL), neutral core (Galβ(1–3)Gal(β1–4)Glc, Galβ(1–6)Gal(β1–4)Glc and LNnT) and fucosylated (LNFP III, 3’-FL and 2’-FL) oligosaccharides.

The current study was based on a previously published animal trial^20^. In that publication, we demonstrated that perinatal prebiotic scFOS supplementation improved adult metabolic health in association with microbiota changes in early and later life, the main hypothesis being that prebiotic scFOS supplementation of the sow during lactation and gestation would modulate the offspring microbiota mainly by direct transfer of the sow microbiota at birth and during lactation^20^. The focus of the current study was to further evaluate the maternal scFOS supplementation-derived changes in milk composition and their relationships with the previously reported infant gut microbiota modifications. The effect of prebiotic supplementation of the maternal diet on MO composition has so far been little explored whereas milk MO concentration is known to be crucial to shape infant gut microbiota, and a better understanding of the factors which drive MO concentration is therefore of great interest. Furthermore, the contribution of sow MOs and lipids to the changes of offspring microbiota has never been studied in the context of maternal prebiotic supplementation.

The integrative approach used in the present study gave a novel insight on the relationships between milk components and faecal microbiota and metabolome in early life.

## Material and methods

### Animals, diets, and experimental design

**Figure 1:**
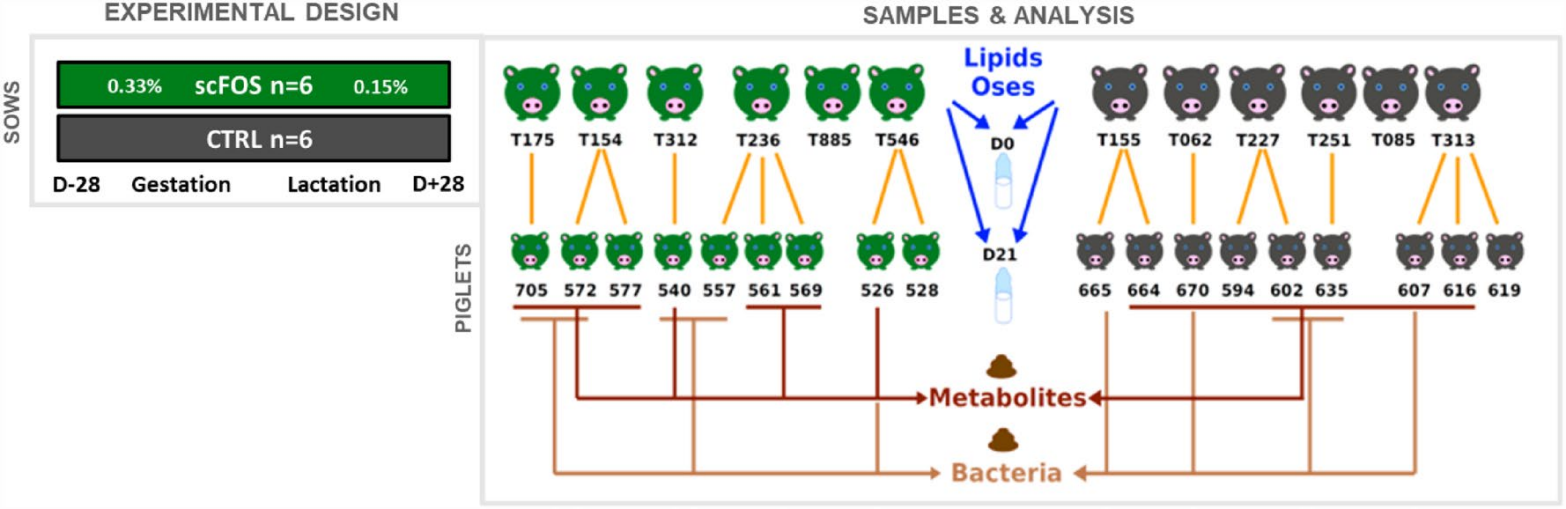
Experimental design, samples and analysis. *CTRL* control group, *scFOS* group supplemented with short chain fructo-oligosaccharides.

The experimental protocol was conducted at INRAE experimental facilities (Pig Physiology and Phenotyping Experimental Facility, Saint-Gilles, France, doi 10.15454/1.5573932732039927E12) and designed in compliance with legislations of the European Union (directive 86 / 609 / EEC) and France (decree 2001-464 29/05/01) for the care and use of laboratory animals (agreement for animal housing number C-35-275-32). The regional Ethics Committee in Animal Experiments of Brittany (France) validated and approved the procedure describes herein (authorization #2016020217308570). Authors complied with the ARRIVE guidelines. Twelve sows (Large White × Landrace) and their piglets [(Large White × Landrace) × Pietrain] from INRAE (Saint-Gilles, France) experimental herd were used. From 28 days before the estimated day of farrowing, sows were fed a standard diet (Cooperl, Lamballe, France), supplemented with either scFOS (Profeed^®^ P95; Beghin-Meiji, France; scFOS group; n = 6) or maltodextrin as a control (Maldex, with a dextrose equivalent of 12; Tereos Starch & Sweeteners Europe, France; CTRL group; n = 6) until weaning of piglets at day 28 (D28). The tested prebiotic was scFOS obtained from sugar beet sucrose through an enzymatic reaction. It is composed of a terminal glucose molecule (G) linked to fructose molecules (F) by a β1–2 bound, with 37 ± 6% 1-kestose (GF2), 47 ± 6% nystose (GF3), and 16 ± 6% 1F-β-fructofuranosyl nystose (GF4), and therefore presents a low degree of polymerization comprised between 3 and 5 (DP 3–5; 95% of scFOS in Profeed^®^ P95; Beghin-Meiji). Sows received 3 kg / d feed during gestation and were fed ad libitum during lactation, resulting in an approximate daily intake of 10 g scFOS over the experimental period (Fig. 1).

Before weaning, piglets had no access to creep feed. Sow body weight and backfat thickness were measured at D-28, D-7 (before parturition), D14 and D28. Body weight of piglets was recorded weekly from birth to weaning. Sows and piglets were monitored daily for food intake, fever and diarrhea. No medication or antibiotic treatment was administered throughout the experiment protocol. At the end of the experiment, piglets were stunned by electronarcosis and killed by exsanguination by a qualified staff member.

### Sample collection

Colostrum and milk were collected from all sows (n = 12) at birth and at day 21 (D21), and faeces were collected from a sub-population of piglets at D21 (n = 18) for different types of analysis described on Fig. 1. Colostrum was collected 1 to 15 h after the onset of parturition and mature milk was collected on D21 postpartum after an intramuscular injection of oxytocin (2 mL, Biocytocine^®^, Laboratoires Biové, Arques, France) to facilitate milking. Colostrum and milk samples were immediately frozen at -20 °C until analysis. Analysis of milk nutritional composition was performed on D21 milk. The PMO profile and concentrations, and the lipidomic profile were measured on colostrum and D21 milk. Faecal samples were collected from a subset of piglets at D21 (n = 5–7/group) to analyse gut microbiota composition^20^ and its fermentative activity (data already published and obtained on the same animal population as the current study^20^), and the metabolome profile. For microbiota and metabolomic analyses, sub-samples of faeces were stored immediately at −80 °C after collection without any treatment, whereas for SCFA analysis, 1 mL 0.5% ortho-phosphoric acid solution per gram of faeces was added, and samples were centrifuged at 1700*g* for 15 min at 4 °C. Supernatants were then stored at −20 °C for later analysis.

### Composition of milk samples

Ash, dry matter, gross energy, proteins, and lipids were assayed as previously described by Loisel et al. (2013)^35^, and carbohydrate contents were calculated as the difference.

### Porcine milk oligosaccharides analysis

The PMOs were extracted, fluorescently derivatised and analysed as described by Albrecht et al*.* (2014) with modifications^29^. Briefly, 50 µL of each sample were diluted ten times with internal standard xylohexaose (X6, O-XHE, Megazyme) and milli-Q water. After homogenization, samples were defatted by centrifugation at 20,000*g* for 20 min at 4 °C. Caseins were precipitated at pH 4.6 by the addition of 15 µL of HCl 0.1 M to the defatted supernatant and centrifugated at 20,000*g* for 20 min at 4 °C. Large peptides were then removed by ultrafiltration (10 kDa Amicon^®^ Ultra 0.5 mL, UFC5010BK, Merck Millipore^®^). The permeates were freeze-dried and suspended in 50 µL milli-Q water. PMOs were fluorescently derivatised via reductive amination with 2-aminobenzamide (2-AB) by the addition of 50 µL of a solution of 2-AB/NaBH_3_CN (sodium cyanoborohydride) in 30% (v/v) acid acetic-dimethylsulfoxide. Samples were incubated at 60 °C for 4 h under orbital agitation. The 2-AB-labelled PMOs were separated by ultra-performance high liquid chromatography (UHPLC) using a Waters BEH glycan column (ACQUITY UPLC Glycan BEH Amide Column, 130 Å, 1.7 µm, 2.1 mm × 150 mm, 186004742, Waters) with fluorescence detection and mass spectrometer (ACQUITY UPLC H-class with FLR and SQD2 detectors, Waters). The mix of solvents used for elution were composed of a 50 mM ammonium formate buffer, pH 4.4 (solvent C) and acetonitrile (solvent B). The elution conditions were 12% solvent C (0–1.5 min), 12–42% solvent C (1.5–30 min) and 12% solvent C (30–40 min). The flow rate was 0.56 mL/min. The injection volume was 6 µL, and the samples were prepared in 80% (v/v) acetonitrile (D12.5 for quantification analyses and D3.125 for mass spectrometry (MS) analyses). The separation temperature was 30 °C. The fluorescence detection wavelengths were λ_ex_ = 330 nm and λ_em_ = 420 nm with a data collection rate of 20 Hz. MS analysis was carried out in positive mode. The source capillary voltage was set to 3 kV and the source temperature was 100 °C. The cone voltage was 80 V. The mass range for MS scan was set to *m/z* 400–2000. A range of external standard Lacto-N-neotetraose (LnNT, OL05765 Carbosynth) was used for quantification. All the data were processed using the Waters Empower3 chromatography workstation software.

### Lipidomic analysis

For lipid extraction, methyl-terbutyl ether was used as the extraction solvent, as fully described by Matyash et al*.* (2008)^36^. Briefly, 1.5 mL of methanol and 5 mL of methyl-terbutyl ether were added to 200 µL of milk sample and vortexed for 1 h. Subsequently, 1.25 mL of deionized water was added to each tube and incubated for 10 min at room temperature, after homogenization. The tubes were then centrifuged at 224*g* for 10 min and the upper organic phase was separated in another tube. The lower phase was washed with 2 mL of a methyl-terbutyl ether/methanol/water mixture (10/3/2.5, v/v/v) and centrifuged as previously for a second extraction. The upper organic phases were pooled, and solvent was evaporated under a stream of nitrogen. Lipid extracts were finally resuspended in 200 µL of a chloroform/methanol/distilled water mixture (60/30/4.5, v/v/v).

The UHPLC separation was performed on a DionexUltiMate 3000 device (Thermo Fisher Scientific, Courtaboeuf, France) using the Accucore C18 column (150 mm × 2.1 mm, 2.6 μm). The column temperature was kept at 45 °C. Mobile phase A contained 10 mmol/L ammonium formate in 60% acetonitrile and 0.1% formic acid, and mobile phase D contained a 10 mmol/L ammonium formate in acetonitrile:propan-2-ol (1/9, v/v) mixture with 0.1% formic acid. The flow rate was 0.4 mL/min. The elution gradient was: 35% D at the beginning, 35% to 60% D for 4 min, 60% to 85% D for 12 min, 85% to 100% D for 21 min, 100% D for 3 min, and 35% D for 4 min. The injection volume was 2 µL. Samples were randomly assigned to the injection table and interspaced (1 of 5) with quality control samples made up of a pool of each sample or solvent for blank. All samples were analysed in a single series.

Samples were analysed using a high-resolution hybrid mass spectrometer Q-Exactive Plus (Thermo Fisher Scientific, Courtaboeuf, France) with electrospray ionization source operating in switching positive and negative ionization polarities. A full scan (*m/z* 250 to 1200) mass spectrum was acquired according to the methodology detailed by Breitkopf et al*.* (2017)^37^. Briefly, the drying temperature was set to 310 °C and the capillary voltage was set to ± 3500 V. Sheath, auxiliary and sweep gas flow rates were 60, 20 and 1 arbitrary units, respectively. The resolving power of the orbitrap mass analyser was set at 35 000 FWHM (Full Width Half Maximum) for the theoretical *m/z* 200 and maximal injection time was set to 200 ms. Full MS spectra were acquired for each sample. Data dependent analysis TOP 15 HCD (Higher Collision induced Dissociation) experiments were used to achieve the structural information of lipid species detected. These spectra were achieved on a pooled sample in separated ionisation modes in order to achieve a structural information for as many lipids detected as possible. Once the data acquired, the resulting spectra were visually inspected to control quality of the signal. The acquired spectra were divided into two groups:

1/ Spectra used for the creation of a data matrix, for use in the XCMS software: the three-dimensional raw data (*m/z*, retention time and ion intensity) obtained from the liquid chromatography-MS analysis were deconvolved in a composite matrix of chromatographic peaks aligned in time and a mass-to-charge ratio (*m/z*) with the intensity of the respective ions. ProteoWizard MS Convert software was used to convert the original data files (*.raw) into more interchangeable formats and centroid mode (*.mzXML) for analysis of both positive and negative spectra. Data processing was performed using the open-source XCMS library as previously described^38^. Once the matrix of raw data was obtained, several filters were applied to eliminate the analytical background and correct the analytical drift. All signals from electronic noise, as well as null samples, were removed manually. Unstable peaks and redundant information were eliminated by using the coefficients of variation of the peaks applied to the control samples.

2/ Spectra used to create a database using LipidSearch software (v4.0, Thermofischer scientific): an in-silico database was built using LipidSearch software, based on the HCD spectra recorded in the samples. This software contains the in-silico database of different lipid families, based on their specific fragmentation pattern and the length of the fatty acid chains. This theoretical fragmentation model was matched to the MS/MS spectra of the milk samples, and the annotations of the various fragmented ions were made based on the *m/z* ratio of the parent ions and the created fragments. In most of the signals, it was possible to identify the corresponding lipid species. Once the lipids in the LipidSearch matrix were identified, the resulting *m/z* and retention time pairs were matched to that of obtained with XCMS. Only the annotated signals have been used to build the final matrix in positive and negative modes. The two matrices were merged while removing the redundancy from the duplicates obtained in the two modes. This curated and annotated matrix was used for statistical analyses.

### Analysis of SCFAs in faecal samples

SCFA assay was performed by gas chromatography in supernatants of ortho-phosphoric acid-treated faeces as previously described^39^.

### Metabolomic analysis

After homogenization of faecal samples, 1 g was vortex mixed with 1 mL of deionized water for 30 s, sonicated for 2 min and vortex mixed second time for 30 s. After a centrifugation at 170,960*g* (Beckman Coulter; Optima L-80XP ultracentrifuge) for 30 min at 4 °C, 1 mL of supernatant (faecal water) was collected and stored at −80 °C. 45 mg of faecal water was extracted with 135 µL of cooled methanol at −20 °C. Samples were vortexed for 1 min and incubated at −20 °C for 30 min. After centrifugation for 15 min at 4 °C and 11,000*g*, 125 µL of supernatant was collected and filtered for 45 min (11,000*g*, 4 °C) through the 10 kDa centrifugal filter tubes. The extracts were dried under the gentle nitrogen stream, resuspended in 250 µL acetonitrile/water (50/50; v/v) mixture. After filtration through the 0.45 µm centrifugal filter tubes for 15 min, samples were placed to the vials with inserts before the LC–MS analysis.

Samples were analyzed using high performance liquid chromatography (HPLC) DionexUltiMate 3000 device (Thermo Fisher Scientific, Courtaboeuf, France), coupled to a high-resolution hybrid mass spectrometer Q-Exactive Plus (Thermo Fisher Scientific, Courtaboeuf, France) with electrospray ionization source (H-ESI II) operating in switching positive and negative ion polarities mode. The chromatographic separation was performed on a binary solvent system using HILIC and reverse phase columns respectively. For the separation of very polar compounds, a SeQuant zic-HILIC column (Merck, 150 mm × 2.1 mm, 5 μm, 200 A) was kept at 25 °C. The mobile phase consisted of a combination of solvent A (100% water, 16 mM ammonium formate) and solvent B (100% acetonitrile 0.1% formic acid) running at 0.25 mL/min flow rate. 5 µL of the sample kept at 4 °C was injected on the column for a separation starting with 2 min isocratic fashion of 97% solvent B. From 2 to 10 min, a linear gradient from 97 to 70% B was applied. From 10 to 15 min, a linear gradient from 70 to 10% B was followed by 2 min isocratic 10% B, 2 min linear gradient from 10 to 97% of B. Equilibration to the initial condition lasted for 4 min. Polar and semi-polar compounds were separated using reverse phase Hypersil Gold C18 (100 mm × 2.1 mm × 1.9 µm) (Thermo Scientific, France) column kept at 40 °C. The flow rate was maintained at 400 µL/mL using 0.1% formic acid solution on water (solvent A) and acetonitrile (solvent B). After 1 min of isocratic flow of 0% B, a 10 min linear gradient of 0% to 100% of B was applied followed by isocratic flow of 100% B for 2 min. After 1 min of linear gradient from 100 to 0% B, a column was equilibrated for 2 min at its initial conditions. Samples were analysed randomly in one analytical batch, interspaced by one pool sample every five samples. One pooled sample was analysed using Higher Collision Energy dissociation experiments in a Data Dependent Analysis workflow for the structural elucidation and identification of metabolites. Ionisation conditions were as follows: spray voltage was kept at ± 3500 V, transfer capillary temperature 320 °C, sheath and auxiliary gas flow rates were 30 and 8 arbitrary units respectively, and the auxiliary gas temperature was 310 °C, S-lens RF voltage was kept at 55 V for the ion transmission. Mass spectra were acquired in the 80–1000 *m/z* range, with resolving power set to 35,000 FWHM for the theoretical *m/z* 200.

Full MS spectra acquired on the HILIC and reverse phase columns in positive and negative ionization modes were pre-processed using XCMS library under R language. Obtained matrices were filtered to eliminate the analytical background and correct any analytical drift and putatively identified using local data base containing information about *m/z*, retention times and fragmentation pattern of more than 800 primary and secondary metabolites. The four matrices were merged while removing the redundancy from the four modes. This curated and annotated matrix was used for statistical analysis.

### Statistics

Data have been processed using RStudio framework (R version 4.1.2) with the help of ade4 package for PCA analysis, mixOmics for multi-bloc integration and ggplot2 to produce specific plots. The amounts of MOs and lipids in sow milk have been considered separately at the two lactation stages through PCA approach as a formal data description, and through unitary statistical tests as an inference assessment of diet effect on each compound. PCAs were applied on scaled values. As MO amounts were not normally distributed, the Wilcoxon test was applied to compare medians between scFOS and CTRL groups as was the Fligner-Killeen test to compare variances.

A log10 transformation was made on lipid dataset to favour the appliance of parametric tests. Before log transformation, some null values were replaced with random values uniformly picked between the minimum value and this minimum value divided by standard deviation of the vector of values tested. By this way, null values mean the smallest amounts rather than missing values due to any technical bias. After a Shapiro-Wilk test to check if lipid values were normally distributed, a student test was applied to compare group means or a Wilcoxon test to compare medians. Tests results were considered according to a 0.05 p-value threshold for significance.

Data from PMOs, lipids and metabolites have been log10 transformed and centred, after null values replacement as described above, for an integrative analysis. The binding of piglet faecal metabolites to sow milk compounds was achieved by selecting the piglet having the maximum values within litter. Multiple batch analysis and cross validations of sparse multi-bloc PLS-DA were used to fix the parameters of the final analysis. This analysis has computed three components, kept at least 10 lipids, 10 metabolites, 5 MOs and included a value 0.1 as block correlations (so-called design matrix).

Scaled data from piglet microbiota, previously described by Le Bourgot et al. (2019)^20^, were further added to the composite dataset in order to project bacteria genus abundances to the clustered image map. As distribution of these amounts were highly heterogeneous, a filter was applied to select genera having a normal distribution (p-value of Shapiro-Wilk test above 0.01).

## Results

### Performances of sows and litters

Sow body weight was not different between groups at the end of gestation and lactation (Table 1). A slight increase in backfat thickness at the end of lactation at D28 was recorded in scFOS group. Litter parameters as total number of piglets per litter, number of born alive and litter body weight were not impacted by maternal scFOS supplementation while a tendency to a higher number of stillborn piglets was observed in scFOS group. However, this number remained very low, less than 1 stillborn on average (Table 1). Maternal scFOS supplementation did not impact piglet body weight during lactation (Supplementary Table 1).

**Table 1:** Performance of sows and litter parameters.

Item	CTRL		scFOS		P-value
	Mean	SEM	Mean	SEM	
Sows performance					
BW D-28, kg	242.5	6.8	247.3	13.5	0.378
BW D-7, kg	266.7	7.4	269.0	13.5	0.441
BW D14, kg	243.3	6.1	245.5	14.2	0.446
BW D28, kg	233.7	6.8	236.2	14.5	0.440
Backfat D-28, mm	16.0	0.7	18.1	1.5	0.113
Backfat D-7, mm	16.6	0.9	18.9	1.5	0.108
Backfat D14, mm	15.5	0.7	16.9	1.4	0.195
Backfat D28, mm	12.6	0.7	14.7	1.3	**0.096**
Litter parameters					
Piglets at birth, n	16.2	0.5	17.0	0.9	0.215
Nb piglets born alive, n	16.0	0.4	16.2	0.7	0.419
Nb stillborn piglets, n	0.2	0.2	0.8	0.4	**0.078**
Litter BW at birth, g	1417.2	114.1	1434.7	78.5	0.451

Mean values ± SEM (n = 6 per group).

*BW* body weight, *D* day with birth as reference day 0, *CTRL* control group, *scFOS* group supplemented with short chain fructo-oligosaccharides.

Significant and trend values (P ≤ 0.10) are in bold.

### Composition of D0-colostrum and D21-milk

#### Macronutrient composition of D21-milk

The analysis of the macronutrient composition was performed on mature milk. The supplementation of sow diet with scFOS did not statistically impact the macronutrient composition of D21-milk, although a tendency to a numerical increase in protein concentration was observed (Table 2).

**Table 2:** Nutritional composition of D21-milk.

Item	CTRL		scFOS		P-value
	Mean	SEM	Mean	SEM	
Total dry matter, g/100 g	18.40	0.39	18.73	0.43	0.289
Ashes, g/100 g	0.68	0.05	0.67	0.02	0.378
Carbohydrates, g/100 g	6.02	0.14	6.03	0.19	0.472
Oligosaccharides, g/100 g	0.068	0.015	0.051	0.012	**0.066**
Lipids, g/100 g	7.32	0.22	7.27	0.28	0.446
Proteins, g/100 g	4.38	0.19	4.77	0.22	0.109
Energy value, kcal/100 g	107.33	2.29	108.67	3.08	0.368

Mean values ± SEM (n = 6 per group).

*CTRL* control group, *scFOS* group supplemented with short chain fructo-oligosaccharides.

Significant and trend values (P ≤ 0.10) are in bold.

#### PMO concentration in colostrum (D0) and mature milk (D21)

Twenty PMOs were identified and quantified in sow milks. These PMOs were divided into 3 classes: acidic, neutral core and fucosylated PMOs. Six acidic PMOs were quantified, including 3’-SL, the most abundant PMO in both D0- and D21-milks (respectively, 2.1 ± 0.8 g/L and 0.35 ± 0.07 g/L), as well as 11 neutral PMOs including 3’-GOS, 6’-GOS, LNnT and LNnH, and 3 fucosylated PMOs including LNFP III, 2’-FL and 3’-FL (Table 3). The total PMO content was in average sixfold higher in D0- than in D21-milk, with a higher PMO variability and diversity on D0 than on D21 (illustrated by PCA on Supplemental Fig. 1 with a higher diameter of ellipses at D0 than at D21). The total PMO content decreased more sharply between D0 and D21 in the scFOS group, leading to a tendency to a lower concentration of PMOs in scFOS group at D21 (P = 0.066). This difference was mainly explained by the significant decrease of neutral core PMOs (P = 0.013). At the individual level, in D0-colostrum, a trend for a higher level of Gal-Gal(β1–4)Glc (Hex3-U1) (neutral PMO) in scFOS group was observed compared to CTRL group (P = 0.065). On D21, the concentration of 3 neutral PMOs was reduced by maternal scFOS supplementation: significantly for a tetrahexose (Gal-Gal-Gal(β1–4)Glc (Hex4-U1)), and as a tendency for two triohexoses, (Galβ(1–3)Gal(β1–4)Glc (3’-GOS), the most abundant neutral core oligosaccharide at D21, and Gal-Gal(β1–4)Glc (Hex3-U1)) (Table 3).

**Table 3:** Milk oligosaccharide composition of sow colostrum (D0) and mature milk (D21) (values are expressed in mg/L).

	D0				P-value (wx)	D21				P-value (wx)
	CTRL		scFOS			CTRL		scFOS		
	Mean	SEM	Mean	SEM		Mean	SEM	Mean	SEM	
Acidic										
3'SL (Neu5Ac(α2–3)Gal(β1–4)Glc)	2087.5	784.7	2385.1	514.6	0.471	353.8	69.8	304.3	90.2	0.298
6’-SL^a^	491.7	450.8	588.5	506.6	0.575	140.3	128.6	86.1	19.6	0.378
Neu5Ac(α2–3)Gal(β1–4)GlcNAc(β1–6)(GlcNAc(β1–3))Gal(β1–4)Glc	13.2	3.3	15.1	5.8	1.000	4.4	1.5	3.2	0.4	0.267
6'SLNnH	9.2	4.3	13.0	7.0	0.230	ND	ND	ND	ND	ND
6'SLNnT	3.1	6.7	3.1	5.4	0.803	ND	ND	ND	ND	ND
3'SLNnT	0.4	0.3	0.6	0.2	0.258	ND	ND	ND	ND	ND
Neutral core										
3'GOS (Galβ(1–3)Gal(β1–4)Glc)	369.9	198.8	410.4	147.6	0.471	40.4	24.8	22.5	4.7	**0.093**
6'GOS (Galβ(1–6)Gal(β1–4)Glc)	56.3	27.4	49.1	26.8	1.000	1.0	0.4	1.1	0.7	0.810
LNnT	52.9	14.1	60.1	23.0	0.810	28.9	13.1	21.9	9.2	0.575
Galα(1–3)Gal(β1–4)Glc	48.2	41.4	38.8	26.4	0.810	5.3	2.4	4.4	1.5	0.471
LNnH	39.4	14.0	43.7	20.3	0.936	6.5	3.8	6.2	3.8	0.936
LNnP I	7.0	2.0	6.5	1.0	0.520	3.7	2.5	2.7	2.2	0.471
Gal–Gal(β1–4)Glc (Hex3-U3)	17.1	10.3	18.7	4.7	0.689	6.7	2.5	5.4	1.9	0.230
Gal–Gal(β1–4)Glc (Hex3-U4)	18.3	5.4	21.2	9.7	0.689	0.6	0.7	0.9	0.8	0.572
Gal–Gal-Gal(β1–4)Glc (Hex4-U1)	6.1	12.5	3.0	3.0	0.630	65.7	12.4	35.9	6.6	**0.005**
Gal–Gal(β1–4)Glc (Hex3-U7)	5.6	3.7	2.3	1.5	0.148	9.8	1.3	6.8	3.3	0.173
Gal–Gal(β1–4)Glc (Hex3-U1)	4.7	1.1	6.3	1.8	**0.065**	2.4	0.4	2.0	0.3	**0.093**
Fucosylated										
LNFP III	27.5	18.4	24.8	14.5	0.810	4.1	1.2	3.8	1.7	0.423
3'FL	22.8	6.3	23.5	6.7	1.000	1.2	1.0	1.0	0.5	0.810
2'FL	11.3	2.3	9.5	0.6	0.127	1.8	1.0	1.6	0.5	0.810
Total										
Acidic	2605.0	737.3	3005.5	904.4	0.471	498.6	116.3	393.6	107.2	0.173
Neutral core	652.9	260.7	684.8	207.0	0.689	175.2	50.3	113.7	17.1	**0.013**
Fucosylated	61.1	15.9	57.8	16.0	0.810	7.1	1.0	6.3	2.0	0.575
Total PMOs	3319.0	679.6	3748.1	1067.5	0.689	680.9	154.1	513.6	117.1	**0.066**

Mean values ± SEM (n = 6 per group).

*CTRL* control group, *scFOS* group supplemented with short chain fructo-oligosaccharides.

^a^Coelution with Neu5Ac(α2–6)Gal(β1–4)GlcNAc (6’-SLN).

Significant and trend values (P ≤ 0.10) are in bold.

#### Lipid profile of D0-colostrum and D21-milk

**Figure 2:**
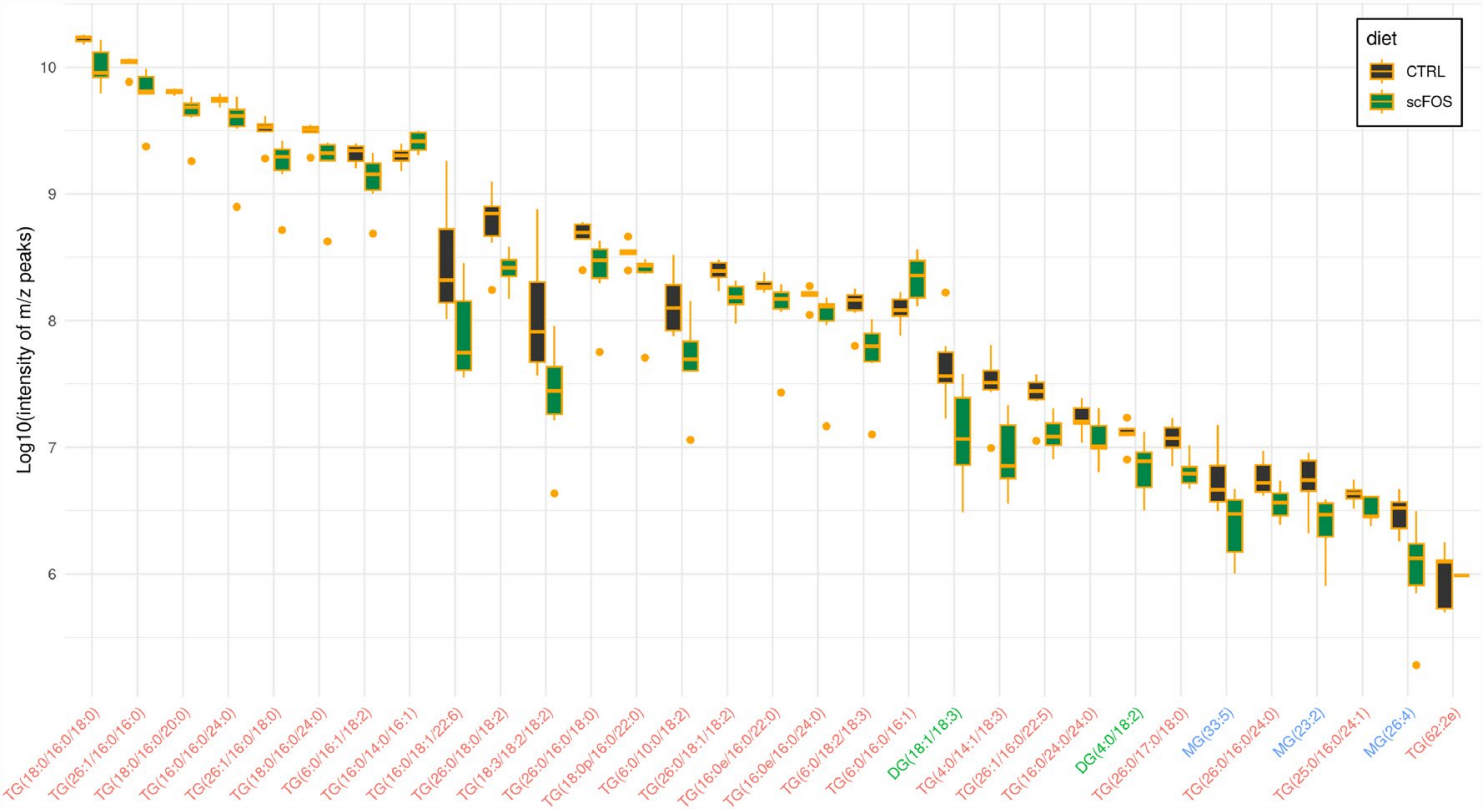
Differential lipidomic profile of milk between control and scFOS groups at day 21. *CTRL* control group, *scFOS* group supplemented with short chain fructo-oligosaccharides, *TG* triglyceride (red), *DG* diglyceride (green), *MG* monoglyceride (blue). Only lipids having significant differences between CTRL and scFOS groups (P-value < 0.05) are presented in descending order of the maximum value measured with the control group.

As for PMO profile, maternal scFOS supplementation impacted lipid profile in D21-milk but not in D0-colostrum. Of the 558 lipids detected belonging to 22 lipid classes (Supplemental Table 2), the maternal scFOS supplementation reduced 29 lipids (24 triglycerides (TG), 2 diglycerides (DG) and 3 monoglycerides (MG)) and increased 2 TG (TG 16:0/14:0/16:1 and TG 6:0/16:0/16:1) (Fig. 2).

The time lactation period discriminated the lipidomic profile, with a higher variability in D0-colostrum *vs* D21-milk as illustrated by PCA where ellipses at D0 and D21 were clearly separated with much higher diameters at D0 compared to D21 (Supplementary Fig. 1). In both D0-colostrum and D21-milk, TG accounted for 80% of lipids while the remaining 20% consisted of polar lipids such as sphingomyelin, glycerophospholipids and ceramides (Supplementary Table 2). Within polar lipids, the proportion of sphingomyelin was higher in D0-colostrum than in D21-milk, where the ceramides dominated (data not shown).

### Gut microbiota composition and SCFA production in D21-piglets

**Figure 3:**
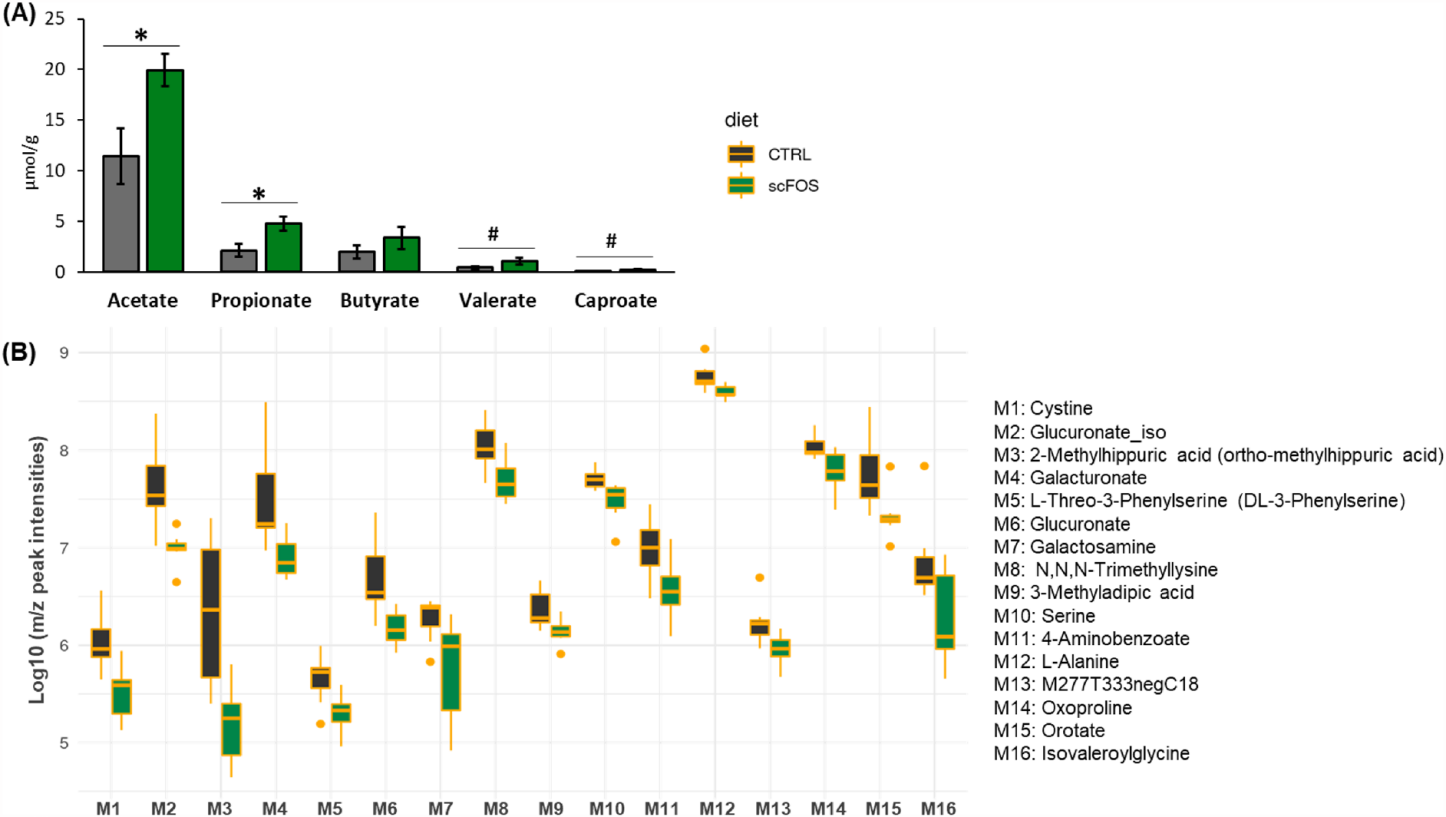
Faecal metabolites of piglets at day 21: (**A**) short-chain fatty acids (SCFA) concentration; (**B**) Differential metabolome profile between control and scFOS groups. *CTRL* control group, *scFOS* group supplemented with short chain fructo-oligosaccharides. Only metabolites having significant differences between CTRL and scFOS groups (P-value < 0.05) are presented.

Results on piglet gut microbiota composition have already been reported in Le Bourgot et al*.* (2019)^20^ using the same animal population as in the current study. Briefly, maternal scFOS supplementation modified piglet faecal microbiota. Among the 10 top important bacterial genera that discriminated the two dietary groups, *Prevotella*, *Bacteroidales-unclassifed*, *RC9_gut_group* and *Fusobacterium* were more abundant in scFOS group whereas *Bacteroides*, *Ruminococcaceae_unclassified, Escherichia-Shigella, Lachnospiraceae_unclassified, Christensenellaceae_unclassified*, and *Clostridiales_unclassified* were less abundant (Supplementary Fig. 2). The SCFA production by the piglet faecal microbiota was also significantly different between groups, with a higher concentration of acetate (P < 0.01), propionate (P < 0.05), valerate (P = 0.06) and caproate (P = 0.07) in scFOS compared to CTRL piglets (Fig. 3A), as reported in a previous publication based on the same population^20^.

### Metabolomic analysis of faecal samples of D21-piglets

The metabolomic approach allowed the annotation of 232 metabolites, among which 16 were significantly reduced in scFOS group compared with CTRL group while none was increased (Fig. 3B). These included amino acids (cystine, serine, L-alanine) and derivatives (DL-3-phenylserine, N6,N6,N6-trimethyl-L-lysine, oxoproline, isovaleroylglycine), uronic acids (glucuronate, iso-glucuronate, galacturonate), hexosamine (galactosamine), fatty acid conjugates (3-methylapidic acid), aminobenzoic acids (4-aminobenzoate) and pyrimidine derivatives (oroate).

### Integrative data analysis of milk components, gut microbiota and faecal metabolites

In order to resume the relationship between D21-sow milk composition and D21-piglet microbial metabolism, an integrative data analysis was performed by sPLS-DA method that selects relevant compounds within the three data blocks (milk lipids, PMOs, and piglet faecal metabolites) participating in the discrimination of the two groups. The sparse method sPLS-DA^40^, formally conceived to classify samples through many variables produced by various -omics analysis, appeared well suited to our dataset, despite the low number of oligosaccharides quantified in sow milk in this integrative analysis. The sPLS-DA analysis revealed a combined effect of scFOS supplementation on amount of lipids, oligosaccharides, and metabolites, without applying a tight constraint to block correlations, as we preferred to configure the analysis to separate at best our two groups of samples. Two lipids, one oligosaccharide and two metabolites were particularly impacted.

**Figure 4:**
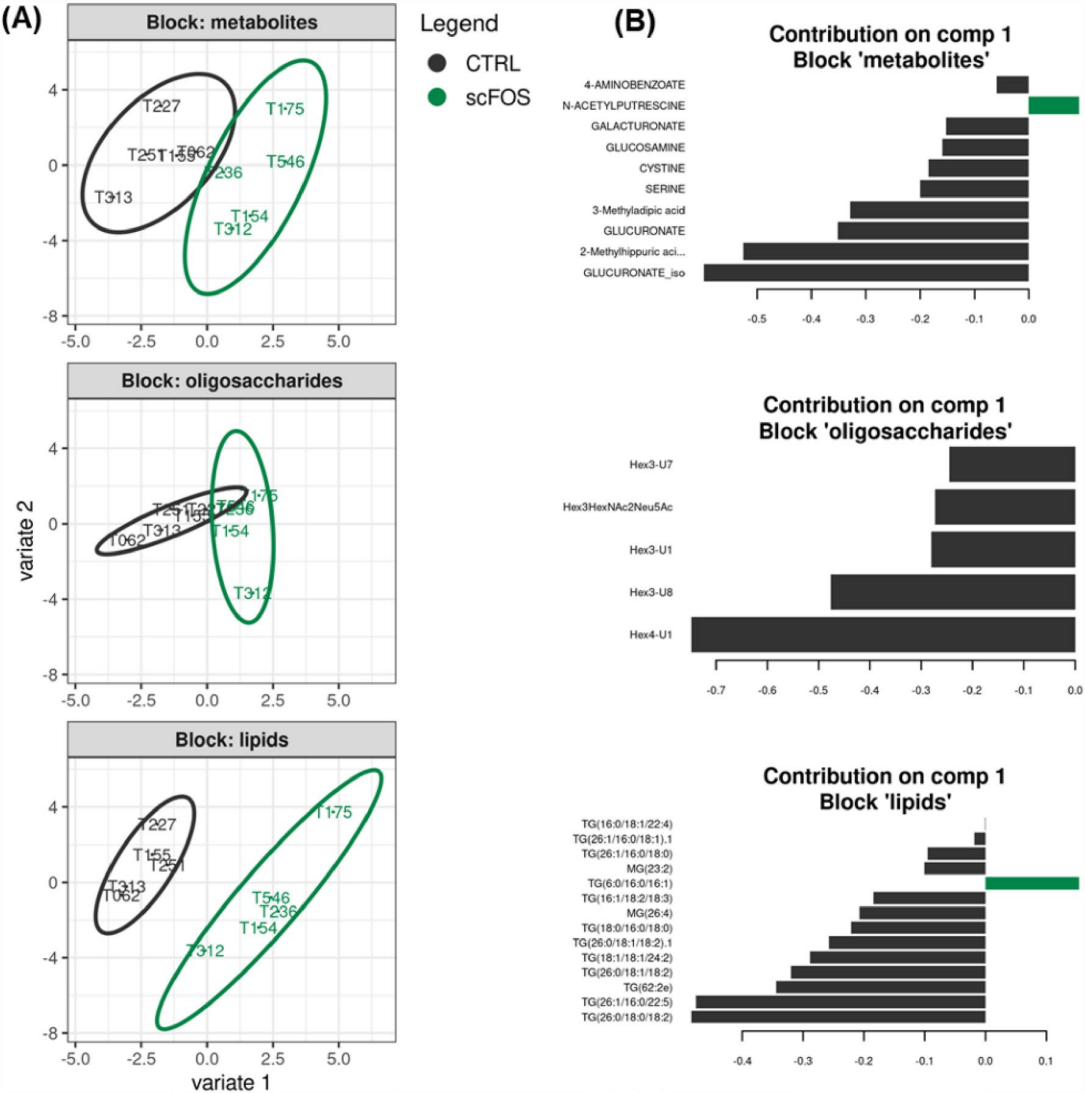
Results of integrative analysis by sPLS-DA on sow D21-milk oligosaccharides and lipids data blocks and on piglet faecal metabolites data block: (**A**) Group discrimination presented by the sample plot for each block of the first two components. Samples are labelled by their corresponding sow identifier; (**B**) potential biomarkers viewed by the variable loadings on the first component. Each bar is coloured according to the group where the related compound is most abundant. *CTRL* control group, *scFOS* group supplemented with short chain fructo-oligosaccharides, *Hex3-U* isomers of Gal-Gal(β1-4)Glc with Hex3-U8 corresponding to Gal(β1–3)Gal(β1–4)Glc (3’-GOS).

The balanced error rate that measured misclassification of individuals, increased from one to three computed components, were respectively 0.09, 0.11 and 0.18, suggesting that the first component gathered the essential information to discriminate the two groups (Fig. 4A). The compounds that contributed most to the first component corresponded to part of the variables, which were significantly changed with scFOS supplementation (Fig. 4B). The increased amounts of sow milk Gal-Gal(β1-4)Glc (labelled as Hex4-U1), Galβ(1–3)Gal(β1–4)Glc (3’-GOS), TG(26:0/18:0/18:2) and TG(26:1/16:0/22:5) and that of piglet faecal glucuronate isoform and 2-methylhippuric acid (2-MHA) shifted individuals towards the CTRL group. At a less contribution level, the increased amounts of the milk lipid TG(6:0/16:0/16:1) and the faecal metabolite N-acetylputrescine were related to the scFOS group. The lipid block size being greater than the other blocks in the present study, the lipid projection on two components appeared as the most efficient to separate scFOS from CTRL sows (Fig. 4A). Although the smallest oligosaccharide block is the less discriminant block in our integrative analysis, the Hex4-U1 oligosaccharide displayed a higher weight on the first component than the TG(26:0/18:0/18:2) and TG(26:1/16:0/22:5) lipids (Fig. 4B). Moreover, high correlations were observed between blocks, especially between lipids and metabolites (Supplementary Fig. 3).

**Figure 5:**
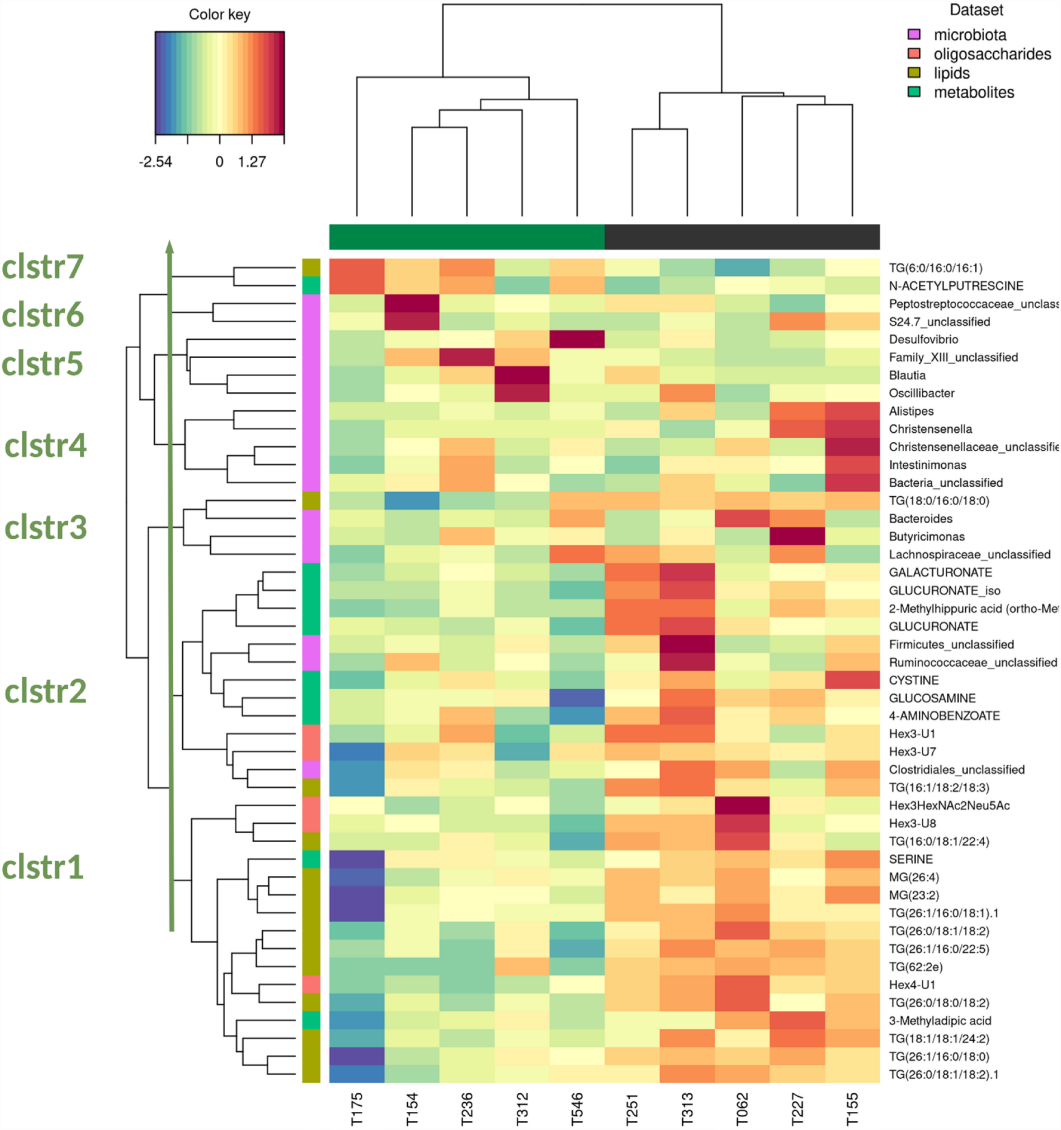
Clustered image map of correlation values between milk components and faecal microbiota and metabolites. Samples are labelled by sow identifiers at bottom and their corresponding diet as top colour tiles (dark green: scFOS, black: CTRL). The top dendrogram corresponds to the sample clustering and the left dendrogram corresponds to the clustering of compounds found in milk and faeces analysis, considering either vertical or horizontal covariance. A reference group of compounds has been arbitrary selected as viewed as the most homogeneous (“clstr1”). Its connection to the left side dendrogram gave a threshold of distance between clusters which is drawn as a green arrow. Cluster numbering starts from bottom to top of the dendrogram. *Clstr* cluster.

A clustered image map was performed to illustrate the correlations between the selected variables resulting from sPLS-DA with the addition of intestinal microbiota composition of piglets (Fig. 5). These microbial data have been included to go further in understanding the link between maternal diet, milk composition and microbial colonization of the infant intestine during the lactation period. A visually well delineated area at the bottom part of the image map gathered the Hex4-U1, TG(26:0/18:0/18:2) and TG(26:1/16:0/22:5) milk components into the cluster 1. The cluster 2 included glucuronate, 2-MHA and some minor contributors of the first sPLS-DA component together with unclassified Firmicutes, Ruminococcaceae and, a little farther, with Clostridiales. The lipid TG(18:0/16:0/16:0), the most abundant lipid in milk that contributed both to the components 1 and 2 of the sPLS-DA (Supplementary Fig. 4), was branched with *Bacteroides* genus (cluster 3). The cluster 7 gathered two components, TG(6:0/16:0/16:1) and N-acetylputrescine, with a higher concentration in scFOS group.

## Discussion

The early-life gut microbiota is a determinant of infant health and is also important for lifelong health. Several perinatal factors contribute to the development and the establishment of infant gut microbiota^41^. In a previous study, we demonstrated that it was possible to modulate the microbiota in suckling piglets by maternal scFOS supplementation during the perinatal period. Indeed, the bacterial composition of scFOS offspring displayed a higher proportion of *Prevotella* genus associated with an increase in total SCFA content, notably acetate, compared with the non-supplemented control group^20^. Such microbiota changes in early life induced long-lasting modulation of intestinal microbiota with beneficial consequences on the host physiology at short and long terms^19,20,42,43^.

The objective of the current study, based on the same animal population, was to evaluate the impact of such scFOS supplementation in the sow diet on milk oligosaccharide and lipid composition and to investigate the relationships between changes in milk composition and piglet gut microbiota modifications in terms of microbial composition (data previously published^20^) and metabolomic profiles. The low number of sows and piglets included in the experimental design, which is a clear limitation of the study, was compensated by rigorous statistical analyses combining the low number of individuals with the large analytical datasets in an integrative approach.

The sow diet prebiotic supplementation had no major impact on the majority of the analysed variables, in line with the relatively low dose of scFOS supplementation (0.33% in gestation and 0.15% in lactation) used in the present study, chosen as such to be applicable in animal nutrition. However, by applying the common alpha threshold of 0.05 on p-values, several significant differences were observed on milk concentrations of total neutral core oligosaccharide, Hexa4-U1 MO and 31 lipids in one hand, and on faecal concentrations of several microbiota genera, 4 short-chain fatty acids and on 16 metabolites in the other hand. In addition, when integrating together all the analysed data through a specific statistical approach, the two groups were clearly separated, highlighting several discriminant variables.

Breast milk composition changes over the lactation period. In the present study, the lactation period clearly impacted PMO profile in terms of quality and quantity. A total of 20 PMOs was detected in sow colostrum samples while only 17 PMOs were quantified in mature milk, 6’-SLNnH, 6’-SLNnT and 3’-SLNnT being not detectable at D21. In the CTRL group, the D21 mature milk was almost 6 times less rich in PMOs than colostrum. The number of PMOs quantified was within the range of PMOs described in literature^29,31–33^. Like human milk, only a small number of PMOs (i.e., 3 to 5 compounds) represented more than 80% of the total PMO amount. In agreement with previous studies reported on porcine milk, 3’-SL was the most abundant PMO both in colostrum and mature milk^31–33,44^. Only two studies^30,45^ found that LNH/LNnH was the most abundant PMO in colostrum. We confirmed the already reported decrease of 3’-SL concentration over lactation^30,32^. A high abundance of 3’-SL may have a favourable effect on health and development of the piglet as a promoter of *Bifidobacterium* growth in the gut^46,47^. However, in the present study, scFOS supplementation of the maternal diet did not significantly impact 3’-SL content in both colostrum and mature milk.

Globally, the maternal scFOS supplementation did not significantly impact the composition of colostrum while moderate differences appeared between the two groups in mature milk in terms of PMO and lipid profiles. Our results on D0-colostrum were highly variable within sow groups and did not point a clear-cut impact of maternal scFOS supplementation at this early stage of lactation. The low number of sows per group as well as the highly variable and rapidly changing composition of colostrum during the first hours following the beginning of the parturition may explain these inconclusive results. Surprisingly, the concentration of total PMOs tended to decrease more sharply between D0 and D21 in the scFOS group than in the CTRL group, leading to a trend lower total PMO concentration in mature milk of scFOS sows than of CTRL sows. More especially, the neutral triohexose (Gal-Gal(β1-4)Glc (Hex3-U1)), which tended to be higher in scFOS group at D0, tended to be lower at D21 as compared to CTRL group. Two other PMOs were reduced by maternal scFOS supplementation at D21: a neutral triohexose named Galβ(1–3)Gal(β1-4)Glc (3’-GOS) as a tendency, and a neutral tetrahexose (Gal-Gal-Gal(β1-4)Glc, Hex4-U1) significantly. Those two PMOs were the third and fourth most abundant PMOs in D21-milk, thereby high contributors to the significant decrease in total neutral core PMOs in scFOS group. To our knowledge, only one study investigated so far the consequences of prebiotic fibre supplementation in maternal diet on the MO content^48^. In the rat model, the authors reported as we do differences between control and supplemented groups, but with increased levels of 2 MOs (one neutral and one acidic) in the supplemented group. The supplementation was much higher than the one we performed (21.5% *vs* 0.33/0.15%), and implied different prebiotic fibres (inulin and oligofructose (ratio 1:1) *vs* scFOS), which may explain these discrepancies. A positive correlation between wholegrains or fruit in the maternal diet (as a proxy of the diet fibre content) and fucosylated MOs, and a negative one with NeuAc MO levels have been described by others^24,25^, but other dietary components than fibres are present in fruit (such as simple sugars) and wholegrains which may account for these effects. A probiotic maternal supplementation during the late stages of pregnancy was reported to modify the oligosaccharide composition of human milk with some HMOs being significantly reduced in the supplemented group^28^. These authors showed that the concentrations of 3’-FL and 3′-SL were significantly higher in the colostrum of mothers who received probiotic supplementation than in control ones, while the total HMO concentration was lower in the probiotic group due to lower levels of difucosyllacto-*N*-hexaose, LNT, LNFP-I, and 6′-SL^28^. In overall, our results confirmed that maternal diet has an impact on total oligosaccharide concentration in milk as well as on specific oligosaccharide level.

In addition to PMOs, a moderate change of the lipid profile was observed in mature milk. Some lipids, mainly triglycerides containing long chain fatty acids (carbon chain ≥ 18), were reduced with scFOS supplementation while only two triglycerides, containing less long chain fatty acids (mainly palmitic acid and derivatives), were increased with scFOS. However, it is worth noting that this is only a small percentage of lipids out of the total number of lipids detected (5.6% of lipids impacted by scFOS diet) and scFOS supplementation did not impact the total concentration of lipids in mature milk. In a previous study using the similar experimental design, we demonstrated that the immune quality of colostrum was significantly improved by maternal scFOS supplementation, while no significant effect was observed in mature milk^19^. Immune parameters of the milk may come from the entero-mammary pathway whereas nutritional composition of mature milk may come from sow metabolism. Indeed, some relationships between maternal body composition and HMO concentrations have been demonstrated during the breastfeeding period in other studies^49,50^. In our study, the scFOS supplementation impacted the sow metabolism at the end of lactation, highlighted by a trend to higher backfat thickness in scFOS group, suggesting that changes in maternal body composition during lactation may qualitatively impact the nutritional composition of the milk. Furthermore, the lipidome and the PMOs were highly correlated in our study (Pearson’s correlation coefficient = 0.85), arguing for a common modulation through sow metabolism. The underlying mechanisms are not established, but as both groups received diets with strictly the same lipid composition (4% lipids mainly from palm oil), differences did not mirror diet differences but may reflect modulation of the sow metabolism, possibly implying intestinal microbiota or insulin signalling in the mammary epithelium. This raises the potential for maternal dietary interventions to improve maternal body composition and health, thereby influencing milk composition to enhance infant growth and development both directly and indirectly (through the infant gut microbiome).

In agreement with our previous microbiota composition analysis, faecal metabolomic profile was modified by scFOS supplementation. However, it is worth stressing that changes were moderate, concerning only a small fraction of the annotated faecal metabolites (only 6.9% of identified metabolite concentrations were modified by maternal scFOS supplementation, and all were reduced).

Our integrative analysis highlighted correlations between milk components and gut microbiota composition and metabolomic profiles. Relationships have already been demonstrated between specific bacteria abundances and MOs^51^. Indeed, the presence of specific enzymatic equipment involved in bacterial HMO catabolism contributes to some bacteria predominance in intestinal lumen. Hence, *Bifidobacterium* spp. was related to human milk with a higher content of sialyllacto-N-tetraose b (LSTb), monofucosyllacto-*N*-hexaose (MFLNH)- III, DSLNT, LNFP I, LNFP III, and LNFP V whereas *Bacteroides* spp. growth benefited from human milk 2’-FL and LNH, illustrating that HMOs directly impact the infant gut microbiota^52^. In vitro studies confirmed that sialyllactose benefited to *Bacteroides* growth in an infant faeces batch culture^53^. Specifically, *Bacteroides* can use as substrates HMOs containing *N*-acetylglucosamine, Gal, Fuc, NeuAc and *N*-acetyl galactosamine^54^. Moreover, HMOs may significantly increase unclassified *Lachnospiraceae* while decreasing *Bacteroides* abundance in young piglets, in a similar way than prebiotic supplementation^55^, in line with our own results. Indeed, in our study, we also observed a reduction of *Bacteroides* abundance at the expense of that of *Prevotella*. This result may be interpreted as positive as infection like rotavirus has been associated with an increase in *Bacteroides* relative abundance^55,56^. Therefore, PMO profile can help establishing gut microbiota in infant piglets^51,57^. Moreover, the high correlation between glucuronate and unclassified Firmicutes, Ruminococcaceae and Clostridiales is in agreement with the expression of enzymes that process glucuronides by these groups of bacteria from Firmicutes phyla, which was reduced in scFOS group^58^. In addition, the fact that faecal N-acetylputrescine was related to the scFOS group in the integrative analysis may suggest a beneficial impact of maternal prebiotic supplementation, as *N*-acetylputrescine was also reported to have a higher abundance in faecal metabolome of breastfed infants and to be positively correlated to *Bifidobacterium* and *Lactobacillus* abundance, which are well-known beneficial bacteria^59^. This is in line with results showing that some strains of *Lactobacillus* increased greatly in D2-piglets whose mothers were supplemented with scFOS during the last week of gestation^60^. Li et al*.* also showed that oroate, which was less in the scFOS group in our study, was in higher abundance in formula-fed infants, and positively associated with *Bacteroides*^59^, suggesting once again a beneficial impact of maternal scFOS supplementation. Altogether, our results highlighted the existing relationship between milk components and faecal microbiota and metabolome. Indeed, the integrative approach gave a novel insight on the link between milk composition, particularly that of oligosaccharides, and the gut microbiota, with the identification of some bacterial metabolites of interest. The addition of prebiotic fibre in the maternal diet during perinatal life may be a beneficial nutritional strategy to orientate the gut microbiota establishment.

As an exploratory study, our processing of the data displays some limitations. The first limitation is the low number of animals per group added to a high variability between animals. Our results highlight therefore potential elements indicating some effects of diet as we explored this small sample size through different strategies: individual statistical tests and an integrative analysis which deals with sparsity of data. A second limitation is the lack of analysis of the milk microbiota. In addition to nutrients, milk also contains numerous bacteria, and it is proposed that a portion of the milk microbiota deriving from the maternal skin or gut may colonize that of the offspring^1,61^. Finally, the low dose of scFOS used in this study to get closer to the doses applicable in porcine and human nutrition, may also explain the moderate impact of the scFOS supplementation on milk composition.

Further investigations are still needed to confirm the results on a larger population and to better understand how breast milk composition can be positively modulated through maternal diet and its relationship with the offspring microbiota to promote short- and long-term growth and health in offspring.

### Supplementary Information

Supplementary Information.

## Data Availability

The datasets generated during the current study are available from the corresponding author on reasonable request.
