# Supplementary material for: Maternal short chain fructo-oligosaccharides supplementation during late gestation and lactation influences milk components and offspring gut metabolome: a pilot study
Source: Sci Rep. 2024 Feb 20;14:4236. doi: 10.1038/s41598-024-54813-3 (PMC10879084; doi:10.1038/s41598-024-54813-3)
Supplement: Supplementary file 1 — Supplementary Information. [file 41598_2024_54813_MOESM1_ESM.pdf]

Maternal short chain fructo-oligosaccharides supplementation during late gestation and lactation influences milk components and offspring gut metabolome: a pilot study

Cindy Le Bourgot[1], Virginie Lollier[2-3], Yoann Richer[2-3], Loric Thoulouze[2-3], Ljubica Svilar[4], Sophie Le Gall[2-3], Sophie Blat[5], Isabelle Le Huërou-Luron[5]

[1]Tereos, Scientific and Regulatory Affairs Department, Moussy-le-Vieux, France

[2]INRAE, UR1268 BIA, F-44300 Nantes, France

[3]INRAE, PROBE research infrastructure, BIBS facility, F-44300 Nantes, France

[4]Cribiom, Centre de Recherche Cardiovasculaire et Nutrition C2VN, UMR INRAE 1260 INSERM 1263 University Aix-Marseille, Marseille, France

[5]Institut NuMeCan, INRAE, INSERM, University Rennes, F-35590 Saint-Gilles, France

SUPPLEMENTARY INFORMATION

Item	CTRL		scFOS		P-value
	Mean	SEM	Mean	SEM	
BW at D0, kg	1.73	0.14	1.72	0.08	0.487
BW at D7, kg	2.78	0.18	2.99	0.14	0.196
BW at D14, kg	5.36	0.28	5.63	0.33	0.276
BW at D21, kg	7.63	0.34	8.04	0.42	0.234
BW at D28, kg	9.67	0.42	9.89	0.57	0.380

Supplementary Table 1. Growth performance of suckling piglets. BW: Body Weight; D: Day with birth as reference day 0; CTRL: control group; scFOS: group supplemented with short chain fructo-oligosaccharides. Mean values ± SEM (n = 8 per group).

Identification		LC-MS results		
Class	Name (RT group)	Lipid ion	m/z	RT
Cer	Cer(d17:1/16:0)_495	Cer(d17:1/16:0)+H	524.50	494.92
Cer	Cer(d17:1/24:1)_658	Cer(d17:1/24:1)+H	634.61	657.84
Cer	Cer(d18:0/16:0)_549	Cer(d18:0/16:0)+H	540.54	548.83
Cer	Cer(d18:0/18:0)_608	Cer(d18:0/18:0)+H	568.57	608.12
Cer	Cer(d18:0/20:0)_663	Cer(d18:0/20:0)+H	596.60	663.23
Cer	Cer(d18:0/22:0)_713	Cer(d18:0/22:0)+H	624.63	713.10
Cer	Cer(d18:0/24:0)_758	Cer(d18:0/24:0)+H	652.66	758.20
Cer	Cer(d18:0/24:1)_702	Cer(d18:0/24:1)+H	650.64	702.35
Cer	Cer(d18:1/14:0)_464	Cer(d18:1/14:0)+H	510.49	464.18
Cer	Cer(d18:1/16:0_499	Cer(d18:1/16:0+O)+H	554.51	499.50
Cer	Cer(d18:1/16:0)_525	Cer(d18:1/16:0)-H	536.50	525.11
Cer	Cer(d18:1/16:0)_525	Cer(d18:1/16:0)+H	538.52	525.55
Cer	Cer(d18:1/17:0)_556	Cer(d18:1/17:0)+H	552.53	556.08
Cer	Cer(d18:1/18:0)_586	Cer(d18:1/18:0)+H	566.55	585.63
Cer	Cer(d18:1/20:0)_642	Cer(d18:1/20:0)+H	594.58	642.02
Cer	Cer(d18:1/22:0)_693	Cer(d18:1/22:0)+H	622.61	693.33
Cer	Cer(d18:1/22:2)_585	Cer(d18:1/22:2)+H	618.58	584.72
Cer	Cer(d18:1/24:0)_739	Cer(d18:1/24:0)-H	648.62	738.90

Cer	Cer(d18:1/24:0)_739	Cer(d18:1/24:0)+H	650.64	739.85
Cer	Cer(d18:1/24:1)_682	Cer(d18:1/24:1)-H	646.61	681.94
Cer	Cer(d18:1/24:1)_682	Cer(d18:1/24:1)+H	648.63	682.53
Cer	Cer(d18:1/24:2)_636	Cer(d18:1/24:2)+H	646.61	635.62
Cer	Cer(d18:1/24:3)_590	Cer(d18:1/24:3)+H	644.60	589.62
Cer	Cer(d18:2/16:0)_469	Cer(d18:2/16:0)+H	536.50	469.46
Cer	Cer(d18:2/18:0)_531	Cer(d18:2/18:0)+H	564.54	530.78
Cer	Cer(d18:2/20:0)_590	Cer(d18:2/20:0)+H	592.57	589.92
Cer	Cer(d18:2/22:0)_644	Cer(d18:2/22:0)+H	620.60	643.52
Cer	Cer(d38:4)_537	Cer(d38:4)+H	588.54	537.21
CerG1	CerG1(d18:1/20:0)_619	CerG1(d18:1/20:0)+H	756.63	619.20
CerG2	CerG2(d30:1)_208	CerG2(d30:1)-H	804.54	207.64
	Cholesterol_483	Cholesterol-H2O+H	369.35	482.74
DG	DG(10:0/18:2)_401	DG(10:0/18:2)+NH4	526.45	400.83
DG	DG(10:0/18:3)_362	DG(10:0/18:3)+NH4	524.43	362.26
DG	DG(10:0/20:4)_387	DG(10:0/20:4)+NH4	550.45	386.51
DG	DG(10:0/22:5)_392	DG(10:0/22:5)+NH4	576.46	391.57
DG	DG(12:0/18:3)_416	DG(12:0/18:3)+NH4	552.46	415.92
DG	DG(14:0/18:3)_350	DG(14:0/18:3)+H	563.47	350.37
DG	DG(14:0/20:4)_499	DG(14:0/20:4)+NH4	606.51	498.59
DG	DG(14:0/22:6)_123	DG(14:0/22:6)+NH4	630.51	122.55
DG	DG(14:0/22:6)_480	DG(14:0/22:6)+NH4	630.51	479.86
DG	DG(15:0)_47	DG(15:0)+NH4	348.27	47.24
DG	DG(15:0/16:0)_593	DG(15:0/16:0)+NH4	572.53	592.81
DG	DG(15:0/18:1)_591	DG(15:0/18:1)+NH4	598.54	590.82
DG	DG(15:0/22:5)_529	DG(15:0/22:5)+NH4	646.54	529.02
DG	DG(16:0/10:0)_448	DG(16:0/10:0)+NH4	502.45	447.67
DG	DG(16:0/14:0)_564	DG(16:0/14:0)+NH4	558.51	564.02
DG	DG(16:0/16:0)_621	DG(16:0/16:0)+NH4	586.54	621.26
DG	DG(16:0/16:0)_621	DG(16:0/16:0)+H	569.51	621.91
DG	DG(16:0/18:1)_619	DG(16:0/18:1)+H	595.53	618.59
DG	DG(16:0/18:1)_619	DG(16:0/18:1)+NH4	612.56	618.65
DG	DG(16:0/18:2)_420	DG(16:0/18:2)+H	593.51	420.28
DG	DG(16:0/18:3)_532	DG(16:0/18:3)+NH4	608.53	531.68
DG	DG(16:0/18:3)_532	DG(16:0/18:3)+H	591.50	531.78
DG	DG(16:0/22:0)_795	DG(16:0/22:0)+NH4	670.63	795.16
DG	DG(16:0/22:4)_597	DG(16:0/22:4)+NH4	662.57	597.29
DG	DG(16:0/24:6)_183	DG(16:0/24:6)+NH4	686.58	183.28
DG	DG(16:0/24:6)_200	DG(16:0/24:6)+NH4	686.58	200.08
DG	DG(16:1/10:0)_393	DG(16:1/10:0)+NH4	500.43	392.55
DG	DG(16:1/14:0)_505	DG(16:1/14:0)+NH4	556.49	504.86
DG	DG(16:1/14:1)_456	DG(16:1/14:1)+NH4	554.48	455.77
DG	DG(16:1/16:1)_508	DG(16:1/16:1)+H	565.48	508.13
DG	DG(16:1/16:1)_513	DG(16:1/16:1)+NH4	582.51	512.97
DG	DG(16:1/18:1)_571	DG(16:1/18:1)+NH4	610.54	571.13
DG	DG(16:1/18:1)_571	DG(16:1/18:1)+H	593.51	571.14

DG	DG(16:1/18:2)_516	DG(16:1/18:2)+NH4	608.53	515.67
DG	DG(16:1/18:2)_516	DG(16:1/18:2)+H	591.50	515.79
DG	DG(16:1/18:3)_358	DG(16:1/18:3)+H	589.48	357.63
DG	DG(16:1/18:3)_475	DG(16:1/18:3)+H	589.48	475.46
DG	DG(16:1/22:6)_130	DG(16:1/22:6)+NH4	656.53	129.82
DG	DG(16:1/24:7)_131	DG(16:1/24:7)+NH4	682.54	130.52
DG	DG(16:2/18:3)_437	DG(16:2/18:3)+NH4	604.49	437.29
DG	DG(17:0/18:1)_645	DG(17:0/18:1)+NH4	626.57	645.43
DG	DG(17:0/22:5)_587	DG(17:0/22:5)+NH4	674.57	586.53
DG	DG(17:1/14:0)_534	DG(17:1/14:0)+NH4	570.51	534.47
DG	DG(17:1/18:1)_598	DG(17:1/18:1)+NH4	624.56	597.94
DG	DG(17:1/18:2)_544	DG(17:1/18:2)+NH4	622.54	543.92
DG	DG(17:1/18:2)_560	DG(17:1/18:2)+NH4	622.54	559.64
DG	DG(17:1/20:5)_489	DG(17:1/20:5)+NH4	644.53	488.88
DG	DG(18:0/16:0)_675	DG(18:0/16:0)+NH4	614.57	674.69
DG	DG(18:0/18:1)_672	DG(18:0/18:1)+NH4	640.59	672.17
DG	DG(18:0/20:3)_639	DG(18:0/20:3)+NH4	664.59	639.05
DG	DG(18:0/20:4)_614	DG(18:0/20:4)+NH4	662.57	614.22
DG	DG(18:0/22:5)_614	DG(18:0/22:5)+NH4	688.59	613.80
DG	DG(18:1/14:0)_563	DG(18:1/14:0)+H	567.50	563.14
DG	DG(18:1/14:0)_563	DG(18:1/14:0)+NH4	584.53	563.14
DG	DG(18:1/18:1)_617	DG(18:1/18:1)+H	621.54	616.83
DG	DG(18:1/18:1)_617	DG(18:1/18:1)+NH4	638.57	617.47
DG	DG(18:1/18:2)_571	DG(18:1/18:2)+NH4	636.56	571.14
DG	DG(18:1/18:2)_571	DG(18:1/18:2)+H	619.53	571.14
DG	DG(18:1/18:2)_590	DG(18:1/18:2)+NH4	636.56	589.92
DG	DG(18:1/18:3)_414	DG(18:1/18:3)+H	617.51	413.97
DG	DG(18:1/20:2)_622	DG(18:1/20:2)+NH4	664.59	621.79
DG	DG(18:1/20:4)_557	DG(18:1/20:4)+H	643.53	556.92
DG	DG(18:1/20:4)_557	DG(18:1/20:4)+NH4	660.56	556.97
DG	DG(18:1/22:2)_671	DG(18:1/22:2)+NH4	692.62	671.14
DG	DG(18:1/22:6)_538	DG(18:1/22:6)+NH4	684.56	538.09
DG	DG(18:2/14:1)_473	DG(18:2/14:1)+NH4	580.49	472.82
DG	DG(18:2/18:2)_525	DG(18:2/18:2)+NH4	634.54	524.68
DG	DG(18:2/18:2)_525	DG(18:2/18:2)+H	617.51	524.68
DG	DG(18:2/18:2)_556	DG(18:2/18:2)+NH4	634.54	556.10
DG	DG(18:3/17:2)_460	DG(18:3/17:2)+NH4	618.51	459.65
DG	DG(18:3/18:2)_484	DG(18:3/18:2)+H	615.50	484.11
DG	DG(18:3/18:2)_484	DG(18:3/18:2)+NH4	632.53	484.33
DG	DG(18:3/18:2)_500	DG(18:3/18:2)+H	615.50	500.32
DG	DG(18:3/18:3)_336	DG(18:3/18:3)+H	613.48	335.90
DG	DG(18:3/18:3)_452	DG(18:3/18:3)+NH4	630.51	452.04
DG	DG(18:3/20:3)_509	DG(18:3/20:3)+NH4	658.54	509.29
DG	DG(18:3/20:4)_471	DG(18:3/20:4)+NH4	656.53	471.15
DG	DG(18:3/20:5)_432	DG(18:3/20:5)+NH4	654.51	432.20
DG	DG(18:3/22:5)_472	DG(18:3/22:5)+NH4	682.54	471.68

DG	DG(18:3/22:6)_453	DG(18:3/22:6)+NH4	680.52	452.65
DG	DG(18:4/16:1)_331	DG(18:4/16:1)+H	587.47	330.61
DG	DG(18:4/16:1)_454	DG(18:4/16:1)+NH4	604.49	454.47
DG	DG(18:4/18:1)_364	DG(18:4/18:1)+H	615.50	364.10
DG	DG(18:4/18:3)_415	DG(18:4/18:3)+NH4	628.49	414.85
DG	DG(19:1/18:1)_641	DG(19:1/18:1)+NH4	652.59	640.88
DG	DG(19:1/18:2)_597	DG(19:1/18:2)+NH4	650.57	597.38
DG	DG(20:2/22:5)_556	DG(20:2/22:5)+NH4	712.59	555.62
DG	DG(20:3/18:2)_535	DG(20:3/18:2)+NH4	660.56	535.45
DG	DG(20:4/20:4)_492	DG(20:4/20:4)+NH4	682.54	491.53
DG	DG(22:3/18:2)_595	DG(22:3/18:2)+NH4	688.59	595.33
DG	DG(22:4/18:2)_544	DG(22:4/18:2)+NH4	686.57	543.57
DG	DG(22:4/18:2)_558	DG(22:4/18:2)+NH4	686.57	557.68
DG	DG(22:5/18:2)_511	DG(22:5/18:2)+NH4	684.56	511.24
DG	DG(24:1/18:2)_717	DG(24:1/18:2)+NH4	720.65	716.68
DG	DG(24:2/18:2)_673	DG(24:2/18:2)+NH4	718.63	673.10
DG	DG(24:4/18:2)_594	DG(24:4/18:2)+NH4	714.60	594.38
DG	DG(31:1)_331	DG(31:1)+H	553.48	330.58
DG	DG(32:3)_472	DG(32:3)+H	563.47	471.94
DG	DG(32:3)_472	DG(32:3)+K	601.42	472.23
DG	DG(33:2)_343	DG(33:2)+H	579.50	343.08
DG	DG(34:3p)_484	DG(34:3p)+Na	597.49	484.26
DG	DG(36:5)_484	DG(36:5)+K	653.45	484.32
DG	DG(38:2)_711	DG(38:2)+NH4	666.60	711.29
DG	DG(4:0/18:2)_238	DG(4:0/18:2)+NH4	442.35	238.13
DG	DG(49:0)_836	DG(49:0)+K	845.74	836.25
DG	DG(50:6)_683	DG(50:6)+H	809.71	683.38
DG	DG(50:6)_701	DG(50:6)+H	809.71	701.34
DG	DG(52:5)_718	DG(52:5)+H	839.75	717.58
DG	DG(52:6)_745	DG(52:6)+H	837.74	744.81
DG	DG(52:7)_683	DG(52:7)+H	835.72	682.52
DG	DG(52:7)_700	DG(52:7)+H	835.72	699.61
DG	DG(52:7)_721	DG(52:7)+H	835.72	720.89
DG	DG(54:3)_755	DG(54:3)+Na	893.80	754.87
DG	DG(54:4)_1010	DG(54:4)+H	869.79	1009.51
DG	DG(54:5)_758	DG(54:5)+H	867.78	758.35
DG	DG(54:7)_726	DG(54:7)+H	863.75	726.39
DG	DG(54:7)_742	DG(54:7)+H	863.75	742.13
DG	DG(55:3)_835	DG(55:3)+K	923.79	835.04
DG	DG(56:5)_764	DG(56:5)+Na	917.80	763.69
DG	DG(56:6)_730	DG(56:6)+Na	915.78	729.60
DG	DG(56:6)_777	DG(56:6)+H	893.80	777.08
DG	DG(56:6)_795	DG(56:6)+H	893.80	795.27
DG	DG(56:7)_721	DG(56:7)+H	891.78	721.06
DG	DG(56:7)_773	DG(56:7)+H	891.78	772.94
DG	DG(56:8)_732	DG(56:8)+H	889.77	731.54

DG	DG(59:5)_831	DG(59:5)+K	975.82	830.87
DG	DG(6:0/18:2)_295	DG(6:0/18:2)+NH4	470.38	294.86
DG	DG(8:0/18:2)_350	DG(8:0/18:2)+NH4	498.42	349.55
dMePE	dMePE(32:2p)_515	dMePE(32:2p)-H	698.51	515.25
FA	FA(22:5)_195	FA(22:5)-H	329.25	195.47
LPC	LPC(16:0)_133	LPC(16:0)+H	496.34	133.29
LPC	LPC(16:0)_150	LPC(16:0)+K	534.30	150.16
LPC	LPC(18:0)_201	LPC(18:0)+HCOO	568.36	201.25
LPC	LPC(18:1)_139	LPC(18:1)+H	522.36	139.18
LPC	LPC(20:3)_203	LPC(20:3)+H	546.35	203.41
LPE	LPE(16:0)_125	LPE(16:0)+H	454.29	124.66
LPE	LPE(16:0)_125	LPE(16:0)-H	452.28	125.26
LPE	LPE(16:0)_79	LPE(16:0)+H	454.29	79.09
LPE	LPE(16:0p)_105	LPE(16:0p)-H	436.28	105.18
LPE	LPE(16:0p)_150	LPE(16:0p)+Na	460.28	150.26
LPE	LPE(16:1)_88	LPE(16:1)-H	450.26	88.32
LPE	LPE(18:1)_131	LPE(18:1)+H	480.31	130.99
LPE	LPE(18:1p)_155	LPE(18:1p)+Na	486.30	154.71
LPE	LPE(18:2)_52	LPE(18:2)-H	476.28	52.47
LPE	LPE(20:3)_186	LPE(20:3)+H	504.31	185.91
LPE	LPE(20:4)_52	LPE(20:4)-H	500.28	52.08
LPE	LPE(20:4)_91	LPE(20:4)-H	500.28	90.94
LPS	LPS(18:0)_135	LPS(18:0)-H	524.30	134.94
LPS	LPS(18:0)_135	LPS(18:0)+H	526.31	135.13
MG	MG(15:2)_52	MG(15:2)+NH4	330.26	51.69
MG	MG(18:2)_147	MG(18:2)+NH4	372.31	146.50
MG	MG(18:3)_52	MG(18:3)+H	353.27	51.78
MG	MG(18:4)_52	MG(18:4)+H	351.25	51.63
MG	MG(23:2)_336	MG(23:2)+NH4	442.39	336.08
MG	MG(23:3)_284	MG(23:3)+NH4	440.37	283.86
MG	MG(26:4)_350	MG(26:4)+NH4	480.40	349.74
MG	MG(29:3)_429	MG(29:3)+NH4	524.47	429.36
MG	MG(29:3)_429	MG(29:3)+H	507.44	429.36
MG	MG(31:3)_479	MG(31:3)+H	535.47	478.61
MG	MG(31:3)_479	MG(31:3)+NH4	552.50	478.73
MG	MG(31:4)_438	MG(31:4)+NH4	550.48	438.36
MG	MG(32:0)_679	MG(32:0)+NH4	572.56	679.07
MG	MG(32:3)_500	MG(32:3)+NH4	566.51	499.55
MG	MG(32:4)_461	MG(32:4)+H	547.47	461.33
MG	MG(32:4)_461	MG(32:4)+NH4	564.50	461.38
MG	MG(33:3)_534	MG(33:3)+H	563.50	533.63
MG	MG(33:3)_534	MG(33:3)+NH4	580.53	533.65
MG	MG(33:4)_486	MG(33:4)+H	561.49	485.86
MG	MG(33:4)_486	MG(33:4)+NH4	578.52	486.25
MG	MG(33:5)_330	MG(33:5)+H	559.47	329.90
MG	MG(34:1)_674	MG(34:1)+NH4	598.58	673.82

MG	MG(36:2)_671	MG(36:2)+NH4	624.59	671.27
MG	MG(36:3)_626	MG(36:3)+NH4	622.58	626.44
MG	MG(36:4)_579	MG(36:4)+NH4	620.56	579.30
MGDG	MGDG(33:1)_203	MGDG(33:1)+HCOO	787.55	203.38
MGDG	MGDG(33:2)_141	MGDG(33:2)-H	739.54	140.94
MGDG	MGDG(35:3)_149	MGDG(35:3)-H	765.55	148.61
OAHFA	OAHFA(18:1/16:0)_515	OAHFA(18:1/16:0)-H	535.47	515.19
OAHFA	OAHFA(18:1/16:1)_480	OAHFA(18:1/16:1)-H	533.46	479.82
OAHFA	OAHFA(18:1/17:1)_508	OAHFA(18:1/17:1)-H	547.47	507.99
OAHFA	OAHFA(18:1/18:1)_533	OAHFA(18:1/18:1)-H	561.49	533.10
OAHFA	OAHFA(18:2/16:1)_438	OAHFA(18:2/16:1)-H	531.44	438.36
OAHFA	OAHFA(18:2/17:1)_464	OAHFA(18:2/17:1)-H	545.46	463.71
OAHFA	OAHFA(18:2/18:1)_486	OAHFA(18:2/18:1)-H	559.47	485.97
OAHFA	OAHFA(18:2/18:2)_447	OAHFA(18:2/18:2)-H	557.46	446.86
OAHFA	OAHFA(20:3/18:1)_495	OAHFA(20:3/18:1)-H	585.49	494.58
PC	PC(16:0/18:1)_635	PC(16:0/18:1)+HCOO	804.57	635.27
PC	PC(18:0/18:2)_574	PC(18:0/18:2)+HCOO	830.59	573.50
PC	PC(18:0p/16:0)_636	PC(18:0p/16:0)+HCOO	790.60	635.79
PC	PC(32:0)_567	PC(32:0)+H	734.57	566.75
PC	PC(32:1)_501	PC(32:1)+H	732.55	501.08
PC	PC(34:2p)_552	PC(34:2p)+H	742.57	552.48
PC	PC(36:1)_635	PC(36:1)+H	788.62	635.10
PE	PE(16:0/16:0)_533	PE(16:0/16:0)-H	690.51	533.16
PE	PE(16:0/16:0)_534	PE(16:0/16:0)+H	692.52	534.40
PE	PE(16:0/16:0)_557	PE(16:0/16:0)+H	692.53	557.00
PE	PE(16:0/16:1)_480	PE(16:0/16:1)-H	688.49	480.46
PE	PE(16:0/18:1)_532	PE(16:0/18:1)-H	716.53	532.21
PE	PE(16:0/18:2)_484	PE(16:0/18:2)-H	714.51	483.91
PE	PE(16:0p/16:0)_567	PE(16:0p/16:0)+H	676.53	566.72
PE	PE(16:0p/16:1)_507	PE(16:0p/16:1)+H	674.51	506.82
PE	PE(16:0p/18:1)_565	PE(16:0p/18:1)+H	702.54	564.81
PE	PE(16:0p/20:4)_501	PE(16:0p/20:4)+H	724.53	501.32
PE	PE(16:0p/20:4)_501	PE(16:0p/20:4)-H	722.51	501.82
PE	PE(16:0p/20:5)_463	PE(16:0p/20:5)-H	720.50	462.62
PE	PE(16:0p/20:5)_463	PE(16:0p/20:5)+H	722.51	462.81
PE	PE(16:0p/22:5)_501	PE(16:0p/22:5)+H	750.54	501.32
PE	PE(16:0p/22:5)_503	PE(16:0p/22:5)-H	748.52	502.56
PE	PE(16:0p/22:6)_482	PE(16:0p/22:6)-H	746.51	482.27
PE	PE(16:0p/22:6)_482	PE(16:0p/22:6)+H	748.53	482.28
PE	PE(17:0/18:2)_511	PE(17:0/18:2)-H	728.52	510.79
PE	PE(18:0/16:0)_592	PE(18:0/16:0)-H	718.54	592.29
PE	PE(18:0/18:1)_590	PE(18:0/18:1)+Na	768.55	590.15
PE	PE(18:0/18:1)_590	PE(18:0/18:1)-H	744.56	590.44
PE	PE(18:0/18:2)_544	PE(18:0/18:2)-H	742.54	544.04
PE	PE(18:0/20:3)_555	PE(18:0/20:3)-H	768.56	555.37
PE	PE(18:0/20:4)_531	PE(18:0/20:4)-H	766.54	531.23

PE	PE(18:0/20:4)_531	PE(18:0/20:4)+H	768.55	531.78
PE	PE(18:0/22:5)_531	PE(18:0/22:5)-H	792.56	531.22
PE	PE(18:0e)_185	PE(18:0e)+H	482.33	185.04
PE	PE(18:0p/18:1)_621	PE(18:0p/18:1)+H	730.57	621.14
PE	PE(18:0p/20:4)_560	PE(18:0p/20:4)+H	752.56	559.61
PE	PE(18:0p/20:4)_561	PE(18:0p/20:4)-H	750.55	560.81
PE	PE(18:1/18:1)_474	PE(18:1/18:1)+Na	766.54	473.83
PE	PE(18:1/18:2)_484	PE(18:1/18:2)-H	740.53	483.95
PE	PE(18:1p/18:1)_561	PE(18:1p/18:1)+H	728.56	561.43
PE	PE(18:1p/18:2)_515	PE(18:1p/18:2)+H	726.54	514.91
PE	PE(18:1p/22:5)_502	PE(18:1p/22:5)+H	776.56	502.04
PE	PE(30:0p)_505	PE(30:0p)-H	646.48	505.38
PE	PE(32:0p)_565	PE(32:0p)-H	674.51	565.35
PE	PE(32:1p)_506	PE(32:1p)-H	672.50	506.16
PE	PE(34:0)_592	PE(34:0)+H	720.55	592.07
PE	PE(34:0p)_623	PE(34:0p)-H	702.55	623.41
PE	PE(34:1)_534	PE(34:1)+Na	740.52	533.53
PE	PE(34:1p)_564	PE(34:1p)-H	700.53	563.51
PE	PE(34:2)_485	PE(34:2)+H	716.52	485.21
PE	PE(36:1)_591	PE(36:1)+H	746.57	590.82
PE	PE(36:1p)_620	PE(36:1p)-H	728.56	620.28
PE	PE(36:2)_544	PE(36:2)+H	744.55	544.06
PE	PE(36:2)_544	PE(36:2)+Na	766.53	544.56
PE	PE(36:2p)_573	PE(36:2p)-H	726.55	572.53
PE	PE(36:3)_485	PE(36:3)+H	742.54	484.88
PE	PE(36:3)_485	PE(36:3)+Na	764.52	484.99
PE	PE(36:3p)_515	PE(36:3p)-H	724.53	515.19
PE	PE(36:4p)_502	PE(36:4p)+Na	746.51	501.57
PE	PE(38:4p)_541	PE(38:4p)-H	750.54	541.26
PE	PE(38:6e)_501	PE(38:6e)+Na	772.52	501.44
PE	PE(39:3)_141	PE(39:3)+H	784.59	141.32
PEt	PEt(15:0/18:1)_511	PEt(15:0/18:1)+H	689.51	511.24
PEt	PEt(15:0/18:1)_538	PEt(15:0/18:1)+H	689.51	538.15
PEt	PEt(15:0/18:2)_492	PEt(15:0/18:2)+H	687.50	491.63
PEt	PEt(15:0/18:3)_453	PEt(15:0/18:3)+H	685.48	452.66
PEt	PEt(52:5)_262	PEt(52:5)+H	947.75	261.63
PEt	PEt(52:7)_202	PEt(52:7)+H	943.72	201.87
PEt	PEt(52:8)_154	PEt(52:8)+Na	963.68	153.70
PEt	PEt(54:8)_205	PEt(54:8)+H	969.73	204.50
PEt	PEt(58:11)_200	PEt(58:11)+H	1019.75	200.14
PG	PG(49:4)_722	953.721226291474	953.72	722.48
PI	PI(16:0/16:0)_445	PI(16:0/16:0)-H	809.52	445.22
PI	PI(16:0/18:1)_446	PI(16:0/18:1)-H	835.54	446.06
PI	PI(18:0/16:0)_499	PI(18:0/16:0)-H	837.55	498.91
PI	PI(18:0/20:4)_448	PI(18:0/20:4)-H	885.55	448.42
PI	PI(18:1/18:1)_457	PI(18:1/18:1)-H	861.55	457.07

PI	PI(18:1/18:2)_407	PI(18:1/18:2)-H	859.54	406.91
PI	PI(38:4)_449	904.591214661882	904.59	448.58
PS	PS(18:0/18:1)_518	PS(18:0/18:1)+H	790.56	518.46
PS	PS(18:0/20:5)_484	PS(18:0/20:5)-H	808.51	483.75
PS	PS(18:0/22:5)_463	PS(18:0/22:5)-H	836.55	463.17
PS	PS(18:1/18:2)_418	PS(18:1/18:2)+H	786.53	417.70
PS	PS(18:2/18:2)_485	PS(18:2/18:2)-H	782.50	484.74
PS	PS(36:2)_480	PS(36:2)+Na	810.53	480.38
PS	PS(36:4p)_515	PS(36:4p)-H	766.50	515.25
PS	PS(38:3)_590	PS(38:3)-H	812.54	590.44
PS	PS(38:4)_544	PS(38:4)-H	810.53	543.83
PS	PS(38:6p)_502	PS(38:6p)-H	790.50	501.73
PS	PS(40:6)_531	PS(40:6)-H	834.53	531.22
PS	PS(47:1)_496	PS(47:1)+K	982.68	495.98
SM	SM(d16:1/18:0)_483	SM(d16:1/18:0)+HCOO	747.57	482.99
SM	SM(d20:1/16:0)_554	SM(d20:1/16:0)+HCOO	775.60	553.59
SM	SM(d32:1)_418	SM(d32:1)+H	675.54	417.90
SM	SM(d33:1)_459	SM(d33:1)+HCOO	733.55	458.57
SM	SM(d34:1)_483	SM(d34:1)+H	703.58	482.88
SM	SM(d34:2)_200	SM(d34:2)+Cl	735.52	200.28
SM	SM(d36:0)_583	SM(d36:0)+H	733.62	582.98
SM	SM(d36:1)_635	SM(d36:1)+H	731.60	634.54
SM	SM(d36:3)_198	SM(d36:3)+Cl	761.54	198.09
SM	SM(d38:0)_692	SM(d38:0)+H	761.65	691.68
SM	SM(d38:0)_705	SM(d38:0)+H	761.65	705.02
SM	SM(d38:1)_622	SM(d38:1)+H	759.64	622.39
SM	SM(d38:4)_552	SM(d38:4)+H	753.59	552.18
SM	SM(d41:1)_718	SM(d41:1)+H	801.69	717.50
SM	SM(d42:2)_668	SM(d42:2)+HCOO	857.68	667.89
SM	SM(d42:2)_668	SM(d42:2)+H	813.68	668.49
SM	SM(d44:1)_791	SM(d44:1)+H	843.73	790.53
SM	SM(d44:2)_753	SM(d44:2)+H	841.71	752.66
TG	TG(10:0/17:1/18:2)_751	TG(10:0/17:1/18:2)+NH4	776.68	750.96
TG	TG(10:0/18:1/18:3)_737	TG(10:0/18:1/18:3)+NH4	788.68	737.22
TG	TG(10:0/18:2/18:3)_699	TG(10:0/18:2/18:3)+NH4	786.66	698.70
TG	TG(10:0/18:2/18:3)_716	TG(10:0/18:2/18:3)+NH4	786.66	715.60
TG	TG(10:0/18:3/18:3)_662	TG(10:0/18:3/18:3)+NH4	784.64	662.28
TG	TG(12:0e/16:0/16:0)_901	TG(12:0e/16:0/16:0)+NH4	754.73	901.48
TG	TG(14:0/14:0/18:3)_780	TG(14:0/14:0/18:3)+NH4	790.69	780.16
TG	TG(14:0/18:2/18:3)_787	TG(14:0/18:2/18:3)+NH4	842.72	787.44
TG	TG(14:0/18:3/18:3)_753	TG(14:0/18:3/18:3)+NH4	840.71	752.84
TG	TG(14:0e/16:0/18:1)_935	TG(14:0e/16:0/18:1)+H	791.75	935.45
TG	TG(14:0e/16:1/16:1)_863	TG(14:0e/16:1/16:1)+NH4	778.73	862.68
TG	TG(14:0p/8:0/18:1)_750	TG(14:0p/8:0/18:1)+NH4	694.63	750.06
TG	TG(15:0/10:0/16:0)_793	TG(15:0/10:0/16:0)+NH4	726.66	793.32
TG	TG(15:0/14:0/16:0)_866	TG(15:0/14:0/16:0)+NH4	782.72	866.22

TG	TG(15:0/16:0/16:0)_901	TG(15:0/16:0/16:0)+NH4	810.75	901.39
TG	TG(15:0/18:2/18:3)_802	TG(15:0/18:2/18:3)+NH4	856.74	802.42
TG	TG(15:1/12:0/16:0)_790	TG(15:1/12:0/16:0)+NH4	752.68	789.84
TG	TG(15:1/14:0/16:0)_828	TG(15:1/14:0/16:0)+NH4	780.71	827.82
TG	TG(15:1/16:0/16:0)_863	TG(15:1/16:0/16:0)+NH4	808.74	863.47
TG	TG(15:1/16:0/16:1)_830	TG(15:1/16:0/16:1)+NH4	806.72	830.26
TG	TG(15:1/16:0/18:1)_865	TG(15:1/16:0/18:1)+NH4	834.75	865.42
TG	TG(15:1/16:0/18:2)_829	TG(15:1/16:0/18:2)+NH4	832.74	829.38
TG	TG(15:1/16:0/24:0)_1008	TG(15:1/16:0/24:0)+NH4	920.86	1008.17
TG	TG(15:1/18:0/22:4)_991	TG(15:1/18:0/22:4)+NH4	912.80	991.43
TG	TG(15:1/18:2/18:3)_778	TG(15:1/18:2/18:3)+NH4	854.72	778.05
TG	TG(15:1/22:5/24:0)_1049	TG(15:1/22:5/24:0)+NH4	994.88	1049.40
TG	TG(15:1/22:5/24:0)_1064	TG(15:1/22:5/24:0)+NH4	994.88	1063.78
TG	TG(16:0/10:0/14:0)_775	TG(16:0/10:0/14:0)+NH4	712.65	775.37
TG	TG(16:0/10:0/17:0)_831	TG(16:0/10:0/17:0)+NH4	754.69	830.62
TG	TG(16:0/10:0/18:1)_808	TG(16:0/10:0/18:1)+NH4	766.69	808.08
TG	TG(16:0/10:0/18:2)_774	TG(16:0/10:0/18:2)+NH4	764.68	774.28
TG	TG(16:0/10:0/18:3)_739	TG(16:0/10:0/18:3)+NH4	762.66	739.23
TG	TG(16:0/10:0/18:3)_740	TG(16:0/10:0/18:3)+Na	767.62	740.47
TG	TG(16:0/10:0/20:4)_760	TG(16:0/10:0/20:4)+NH4	788.68	760.26
TG	TG(16:0/12:0/14:0)_812	TG(16:0/12:0/14:0)+NH4	740.68	812.28
TG	TG(16:0/12:0/20:4)_799	TG(16:0/12:0/20:4)+NH4	816.71	799.08
TG	TG(16:0/14:0/14:0)_847	TG(16:0/14:0/14:0)+NH4	768.71	846.94
TG	TG(16:0/14:0/16:0)_903	TG(16:0/14:0/16:0)+NH4	796.74	903.09
TG	TG(16:0/14:0/16:1)_843	TG(16:0/14:0/16:1)+NH4	794.72	842.71
TG	TG(16:0/14:0/18:3)_822	TG(16:0/14:0/18:3)+NH4	818.72	821.64
TG	TG(16:0/16:0/16:0)_925	TG(16:0/16:0/16:0)+NH4	824.77	924.99
TG	TG(16:0/16:0/16:1)_781	TG(16:0/16:0/16:1)+NH4	822.75	780.66
TG	TG(16:0/16:0/17:0)_940	TG(16:0/16:0/17:0)+NH4	838.79	940.44
TG	TG(16:0/16:0/17:1)_900	TG(16:0/16:0/17:1)+NH4	836.77	899.97
TG	TG(16:0/16:0/18:1)_742	TG(16:0/16:0/18:1)+Na	855.74	742.26
TG	TG(16:0/16:0/18:3)_858	TG(16:0/16:0/18:3)+NH4	846.75	858.17
TG	TG(16:0/16:0/23:0)_1049	TG(16:0/16:0/23:0)+NH4	922.88	1049.45
TG	TG(16:0/16:0/24:0)_1067	TG(16:0/16:0/24:0)+NH4	936.90	1067.44
TG	TG(16:0/16:1/18:1)_703	TG(16:0/16:1/18:1)+Na	853.73	702.56
TG	TG(16:0/16:1/18:1)_873	TG(16:0/16:1/18:1)+NH4	848.77	872.86
TG	TG(16:0/16:1/18:1)_889	TG(16:0/16:1/18:1)+NH4	848.77	888.72
TG	TG(16:0/16:1/18:2)_838	TG(16:0/16:1/18:2)+NH4	846.75	837.95
TG	TG(16:0/16:1/18:3)_705	TG(16:0/16:1/18:3)+H	827.71	704.96
TG	TG(16:0/16:1/18:3)_836	TG(16:0/16:1/18:3)+NH4	844.74	836.16
TG	TG(16:0/17:0/18:1)_937	TG(16:0/17:0/18:1)+NH4	864.80	936.82
TG	TG(16:0/17:0/18:3)_864	TG(16:0/17:0/18:3)+NH4	860.77	863.91
TG	TG(16:0/17:1/18:1)_897	TG(16:0/17:1/18:1)+NH4	862.79	896.99
TG	TG(16:0/17:1/20:4)_956	TG(16:0/17:1/20:4)+NH4	884.78	955.61
TG	TG(16:0/17:2/18:2)_964	TG(16:0/17:2/18:2)+NH4	858.76	964.32
TG	TG(16:0/18:1/18:1)_1077	TG(16:0/18:1/18:1)+Na	881.76	1076.79

TG	TG(16:0/18:1/18:1)_926	TG(16:0/18:1/18:1)+NH4	876.80	925.76
TG	TG(16:0/18:1/18:1)_942	TG(16:0/18:1/18:1)+NH4	876.80	942.17
TG	TG(16:0/18:1/18:3)_840	TG(16:0/18:1/18:3)+NH4	872.77	840.48
TG	TG(16:0/18:1/18:3)_870	TG(16:0/18:1/18:3)+NH4	872.77	870.29
TG	TG(16:0/18:1/19:5)_759	TG(16:0/18:1/19:5)+Na	887.71	758.74
TG	TG(16:0/18:1/20:2)_691	TG(16:0/18:1/20:2)+Na	907.77	691.34
TG	TG(16:0/18:1/21:6)_754	TG(16:0/18:1/21:6)+Na	913.72	754.36
TG	TG(16:0/18:1/22:4)_759	TG(16:0/18:1/22:4)+H	909.79	758.50
TG	TG(16:0/18:1/22:5)_727	TG(16:0/18:1/22:5)+H	907.78	727.41
TG	TG(16:0/18:1/22:6)_691	TG(16:0/18:1/22:6)+H	905.76	691.36
TG	TG(16:0/18:1/24:1)_1016	TG(16:0/18:1/24:1)+NH4	960.90	1015.88
TG	TG(16:0/18:1/24:1)_1029	TG(16:0/18:1/24:1)+NH4	960.89	1028.96
TG	TG(16:0/18:2/18:3)_824	TG(16:0/18:2/18:3)+NH4	870.75	824.20
TG	TG(16:0/18:2/21:6)_766	TG(16:0/18:2/21:6)+Na	911.71	765.68
TG	TG(16:0/18:2/22:5)_835	TG(16:0/18:2/22:5)+NH4	922.79	835.04
TG	TG(16:0/18:2/22:6)_648	TG(16:0/18:2/22:6)+H	903.75	648.09
TG	TG(16:0/18:2/22:6)_825	TG(16:0/18:2/22:6)+NH4	920.77	825.26
TG	TG(16:0/18:2/24:1)_982	TG(16:0/18:2/24:1)+NH4	958.88	982.18
TG	TG(16:0/18:3/18:3)_794	TG(16:0/18:3/18:3)+NH4	868.74	794.41
TG	TG(16:0/20:4/24:0)_1017	TG(16:0/20:4/24:0)+NH4	984.90	1017.17
TG	TG(16:0/24:0/24:0)_1195	TG(16:0/24:0/24:0)+NH4	1049.02	1194.75
TG	TG(16:0/8:0/18:1)_771	TG(16:0/8:0/18:1)+NH4	738.66	770.92
TG	TG(16:0/8:0/20:4)_706	TG(16:0/8:0/20:4)+NH4	760.65	705.92
TG	TG(16:0e/10:0/16:0)_865	TG(16:0e/10:0/16:0)+NH4	726.70	865.08
TG	TG(16:0e/10:0/16:1)_825	TG(16:0e/10:0/16:1)+NH4	724.68	825.28
TG	TG(16:0e/14:0/16:0)_940	TG(16:0e/14:0/16:0)+NH4	782.76	940.26
TG	TG(16:0e/14:0/16:0)_940	TG(16:0e/14:0/16:0)+H	765.73	940.31
TG	TG(16:0e/16:0/16:0)_979	TG(16:0e/16:0/16:0)+NH4	810.79	978.59
TG	TG(16:0e/16:0/16:0)_979	TG(16:0e/16:0/16:0)+H	793.76	978.66
TG	TG(16:0e/16:0/18:1)_973	TG(16:0e/16:0/18:1)+H	819.78	973.15
TG	TG(16:0e/16:0/18:1)_973	TG(16:0e/16:0/18:1)+NH4	836.81	973.15
TG	TG(16:0e/16:0/22:0)_1089	TG(16:0e/16:0/22:0)+NH4	894.88	1088.51
TG	TG(16:0e/16:0/22:1)_1045	TG(16:0e/16:0/22:1)+NH4	892.87	1045.20
TG	TG(16:0e/16:0/24:0)_1122	TG(16:0e/16:0/24:0)+NH4	922.92	1122.14
TG	TG(16:0e/18:1/18:1)_967	TG(16:0e/18:1/18:1)+H	845.80	967.03
TG	TG(16:0e/18:1/18:1)_967	TG(16:0e/18:1/18:1)+NH4	862.82	967.84
TG	TG(16:0e/18:1/22:2)_1004	TG(16:0e/18:1/22:2)+NH4	916.87	1004.26
TG	TG(16:0e/18:2/18:2)_901	TG(16:0e/18:2/18:2)+NH4	858.79	901.44
TG	TG(16:0e/18:2/22:5)_903	TG(16:0e/18:2/22:5)+NH4	908.81	903.26
TG	TG(16:0e/18:2/24:1)_1034	TG(16:0e/18:2/24:1)+NH4	944.90	1034.44
TG	TG(16:0p/16:1/16:1)_861	TG(16:0p/16:1/16:1)+NH4	804.74	860.90
TG	TG(16:1/10:0/16:1)_733	TG(16:1/10:0/16:1)+NH4	736.65	732.88
TG	TG(16:1/14:0/18:1)_841	TG(16:1/14:0/18:1)+NH4	820.74	841.05
TG	TG(16:1/14:0/18:3)_779	TG(16:1/14:0/18:3)+NH4	816.71	779.49
TG	TG(16:1/14:1/17:1)_800	TG(16:1/14:1/17:1)+NH4	804.71	800.36
TG	TG(16:1/18:1/18:1)_873	TG(16:1/18:1/18:1)+NH4	874.78	872.80

TG	TG(16:1/18:2/18:3)_783	TG(16:1/18:2/18:3)+NH4	868.74	782.52
TG	TG(16:1/18:2/22:6)_614	TG(16:1/18:2/22:6)+H	901.73	614.12
TG	TG(16:1/18:2/22:6)_638	TG(16:1/18:2/22:6)+H	901.73	637.50
TG	TG(16:1/18:3/18:3)_752	TG(16:1/18:3/18:3)+NH4	866.72	752.24
TG	TG(17:0/18:1/18:2)_900	TG(17:0/18:1/18:2)+NH4	888.80	899.55
TG	TG(17:0/18:1/22:5)_869	TG(17:0/18:1/22:5)+NH4	938.82	869.28
TG	TG(18:0/16:0/16:0)_962	TG(18:0/16:0/16:0)+NH4	852.80	961.87
TG	TG(18:0/16:0/18:0)_998	TG(18:0/16:0/18:0)+NH4	880.83	998.00
TG	TG(18:0/16:0/18:1)_941	TG(18:0/16:0/18:1)+NH4	878.81	940.75
TG	TG(18:0/16:0/18:1)_956	TG(18:0/16:0/18:1)+NH4	878.82	956.25
TG	TG(18:0/16:0/19:0)_1015	TG(18:0/16:0/19:0)+NH4	894.85	1014.91
TG	TG(18:0/16:0/20:0)_1034	TG(18:0/16:0/20:0)+NH4	908.86	1033.54
TG	TG(18:0/16:0/22:4)_935	TG(18:0/16:0/22:4)+NH4	928.83	934.55
TG	TG(18:0/16:0/24:0)_1101	TG(18:0/16:0/24:0)+NH4	964.93	1100.88
TG	TG(18:0/17:0/18:1)_974	TG(18:0/17:0/18:1)+NH4	892.83	973.57
TG	TG(18:0/18:1/18:1)_949	TG(18:0/18:1/18:1)+NH4	904.83	949.14
TG	TG(18:0/18:1/18:3)_758	TG(18:0/18:1/18:3)+H	883.78	757.71
TG	TG(18:0/18:1/18:3)_891	TG(18:0/18:1/18:3)+NH4	900.80	890.70
TG	TG(18:0/18:1/22:6)_892	TG(18:0/18:1/22:6)+NH4	950.82	892.09
TG	TG(18:0e/16:0/16:0)_1017	TG(18:0e/16:0/16:0)+NH4	838.82	1016.92
TG	TG(18:0e/16:0/18:1)_1010	TG(18:0e/16:0/18:1)+NH4	864.84	1009.70
TG	TG(18:0e/16:0/24:0)_1155	TG(18:0e/16:0/24:0)+NH4	950.95	1154.90
TG	TG(18:0e/18:1/18:1)_1005	TG(18:0e/18:1/18:1)+NH4	890.85	1005.14
TG	TG(18:0e/18:2/18:2)_929	TG(18:0e/18:2/18:2)+NH4	886.82	929.31
TG	TG(18:0p/10:0/16:0)_861	TG(18:0p/10:0/16:0)+NH4	752.71	860.65
TG	TG(18:0p/14:0/16:0)_935	TG(18:0p/14:0/16:0)+NH4	808.78	934.92
TG	TG(18:0p/14:0/16:1)_901	TG(18:0p/14:0/16:1)+NH4	806.76	900.56
TG	TG(18:0p/16:0/18:2)_932	TG(18:0p/16:0/18:2)+NH4	860.81	932.23
TG	TG(18:0p/16:0/18:2)_932	TG(18:0p/16:0/18:2)+H	843.78	932.40
TG	TG(18:0p/16:0/22:0)_1079	TG(18:0p/16:0/22:0)+NH4	920.90	1078.90
TG	TG(18:0p/16:0/22:1)_1039	TG(18:0p/16:0/22:1)+NH4	918.89	1038.98
TG	TG(18:0p/16:0/24:0)_1113	TG(18:0p/16:0/24:0)+NH4	948.93	1113.42
TG	TG(18:0p/18:3/24:0)_1032	TG(18:0p/18:3/24:0)+NH4	970.92	1031.87
TG	TG(18:1/17:1/18:2)_1001	TG(18:1/17:1/18:2)+NH4	886.79	1001.28
TG	TG(18:1/17:1/18:2)_862	TG(18:1/17:1/18:2)+NH4	886.79	861.72
TG	TG(18:1/18:1/22:2)_930	TG(18:1/18:1/22:2)+NH4	956.86	930.40
TG	TG(18:1/18:1/22:2)_944	TG(18:1/18:1/22:2)+NH4	956.86	943.94
TG	TG(18:1/18:1/22:4)_762	TG(18:1/18:1/22:4)+H	935.81	762.16
TG	TG(18:1/18:1/24:2)_979	TG(18:1/18:1/24:2)+NH4	984.90	978.70
TG	TG(18:1/18:1/24:2)_992	TG(18:1/18:1/24:2)+NH4	984.90	991.88
TG	TG(18:1/18:2/18:3)_709	TG(18:1/18:2/18:3)+H	879.74	708.84
TG	TG(18:1/18:2/18:3)_818	TG(18:1/18:2/18:3)+NH4	896.77	817.68
TG	TG(18:1/18:2/20:2)_895	TG(18:1/18:2/20:2)+NH4	926.82	895.37
TG	TG(18:1/18:2/22:2)_902	TG(18:1/18:2/22:2)+NH4	954.84	901.73
TG	TG(18:1/18:2/22:3)_864	TG(18:1/18:2/22:3)+NH4	952.82	864.20
TG	TG(18:1/18:2/22:3)_900	TG(18:1/18:2/22:3)+NH4	952.83	899.63

TG	TG(18:1/18:2/22:4)_864	TG(18:1/18:2/22:4)+NH4	950.82	864.16
TG	TG(18:1/18:2/22:5)_833	TG(18:1/18:2/22:5)+NH4	948.80	832.71
TG	TG(18:1/18:2/24:2)_944	TG(18:1/18:2/24:2)+NH4	982.88	943.90
TG	TG(18:1/20:4/22:5)_823	TG(18:1/20:4/22:5)+NH4	972.80	823.09
TG	TG(18:1/22:5/24:1)_967	TG(18:1/22:5/24:1)+NH4	1034.91	967.10
TG	TG(18:1p/16:0/16:0)_938	TG(18:1p/16:0/16:0)+NH4	834.79	938.05
TG	TG(18:1p/16:0/18:3)_869	TG(18:1p/16:0/18:3)+NH4	856.77	868.68
TG	TG(18:2/17:1/18:2)_830	TG(18:2/17:1/18:2)+NH4	884.77	830.31
TG	TG(18:2/18:2/18:2)_837	TG(18:2/18:2/18:2)+NH4	896.77	836.66
TG	TG(18:3/17:1/18:2)_801	TG(18:3/17:1/18:2)+NH4	882.75	800.69
TG	TG(18:3/17:2/18:2)_770	TG(18:3/17:2/18:2)+NH4	880.74	769.84
TG	TG(18:3/18:2/18:2)_647	TG(18:3/18:2/18:2)+H	877.73	647.20
TG	TG(18:3/18:2/18:2)_788	TG(18:3/18:2/18:2)+NH4	894.75	787.90
TG	TG(18:3/18:2/20:3)_802	TG(18:3/18:2/20:3)+NH4	920.77	802.44
TG	TG(18:3/18:2/20:4)_775	TG(18:3/18:2/20:4)+NH4	918.75	775.27
TG	TG(18:3/18:2/22:5)_773	TG(18:3/18:2/22:5)+NH4	944.77	772.65
TG	TG(18:3/18:2/22:6)_759	TG(18:3/18:2/22:6)+NH4	942.75	759.24
TG	TG(19:1/16:0/18:1)_933	TG(19:1/16:0/18:1)+NH4	890.82	933.19
TG	TG(19:1/18:0/18:1)_969	TG(19:1/18:0/18:1)+NH4	918.85	968.96
TG	TG(19:1/18:2/18:2)_864	TG(19:1/18:2/18:2)+NH4	912.80	863.83
TG	TG(20:0e/10:0/10:0)_829	TG(20:0e/10:0/10:0)+NH4	698.67	829.00
TG	TG(20:0e/16:0/16:0)_1054	TG(20:0e/16:0/16:0)+NH4	866.85	1053.56
TG	TG(20:0p/16:0/24:0)_1146	TG(20:0p/16:0/24:0)+NH4	976.96	1146.13
TG	TG(20:0p/21:1/21:1)_1108	TG(20:0p/21:1/21:1)+NH4	1000.96	1107.93
TG	TG(20:1/18:1/18:1)_943	TG(20:1/18:1/18:1)+NH4	930.85	942.90
TG	TG(20:1/18:1/18:2)_903	TG(20:1/18:1/18:2)+NH4	928.83	903.22
TG	TG(20:2/18:2/18:2)_866	TG(20:2/18:2/18:2)+NH4	924.80	866.18
TG	TG(20:3/17:1/18:2)_835	TG(20:3/17:1/18:2)+NH4	910.79	834.68
TG	TG(20:5/18:2/22:5)_761	TG(20:5/18:2/22:5)+NH4	968.77	760.72
TG	TG(22:5/18:2/18:2)_801	TG(22:5/18:2/18:2)+NH4	946.78	801.37
TG	TG(22:5/18:2/20:4)_790	TG(22:5/18:2/20:4)+NH4	970.78	789.83
TG	TG(24:0/18:2/18:3)_970	TG(24:0/18:2/18:3)+NH4	982.88	969.72
TG	TG(24:1/18:2/22:5)_932	TG(24:1/18:2/22:5)+NH4	1032.90	931.96
TG	TG(24:3/18:1/18:2)_897	TG(24:3/18:1/18:2)+NH4	980.86	897.48
TG	TG(24:4/16:0/18:1)_941	TG(24:4/16:0/18:1)+NH4	954.85	940.73
TG	TG(24:4/18:1/18:2)_898	TG(24:4/18:1/18:2)+NH4	978.85	897.56
TG	TG(24:5/18:2/18:2)_831	TG(24:5/18:2/18:2)+NH4	974.82	830.87
TG	TG(25:0/16:0/16:0)_1084	TG(25:0/16:0/16:0)+NH4	950.91	1083.66
TG	TG(25:0/16:0/18:0)_1117	TG(25:0/16:0/18:0)+NH4	978.94	1116.99
TG	TG(25:0/16:0/18:3)_1006	TG(25:0/16:0/18:3)+NH4	972.90	1005.98
TG	TG(25:0/16:0/24:1)_1173	TG(25:0/16:0/24:1)+NH4	1061.02	1173.14
TG	TG(25:0/18:1/18:1)_1073	TG(25:0/18:1/18:1)+NH4	1002.94	1072.75
TG	TG(25:0/18:1/18:2)_1039	TG(25:0/18:1/18:2)+NH4	1000.93	1039.34
TG	TG(25:0/18:2/18:2)_1008	TG(25:0/18:2/18:2)+NH4	998.91	1007.89
TG	TG(25:1/16:0/16:0)_1043	TG(25:1/16:0/16:0)+NH4	948.90	1043.46
TG	TG(25:1/16:0/18:0)_1078	TG(25:1/16:0/18:0)+NH4	976.93	1077.73

TG	TG(25:1/16:0/18:1)_1036	TG(25:1/16:0/18:1)+NH4	974.91	1036.49
TG	TG(26:0/16:0/18:0)_1133	TG(26:0/16:0/18:0)+NH4	992.96	1133.38
TG	TG(26:0/16:0/24:0)_1224	TG(26:0/16:0/24:0)+NH4	1077.05	1224.04
TG	TG(26:0/17:0/18:0)_1149	TG(26:0/17:0/18:0)+NH4	1006.97	1149.18
TG	TG(26:0/17:0/18:1)_1111	TG(26:0/17:0/18:1)+NH4	1004.96	1111.00
TG	TG(26:0/18:0/18:0)_1165	TG(26:0/18:0/18:0)+NH4	1020.99	1164.95
TG	TG(26:0/18:0/18:1)_1127	TG(26:0/18:0/18:1)+NH4	1018.97	1127.17
TG	TG(26:0/18:0/18:2)_1089	TG(26:0/18:0/18:2)+NH4	1016.96	1089.11
TG	TG(26:0/18:0/20:3)_1078	TG(26:0/18:0/20:3)+NH4	1042.97	1078.12
TG	TG(26:0/18:0/24:0)_1252	TG(26:0/18:0/24:0)+NH4	1105.08	1251.72
TG	TG(26:0/18:1/18:2)_1026	TG(26:0/18:1/18:2)+NH4	1014.93	1025.55
TG	TG(26:0/18:1/18:2)_1045	TG(26:0/18:1/18:2)+NH4	1014.94	1044.51
TG	TG(26:0/18:1/18:2)_1058	TG(26:0/18:1/18:2)+NH4	1014.94	1057.58
TG	TG(26:0/18:2/18:3)_1010	TG(26:0/18:2/18:3)+NH4	1010.91	1010.43
TG	TG(26:1/16:0/16:0)_1061	TG(26:1/16:0/16:0)+NH4	962.91	1060.73
TG	TG(26:1/16:0/18:0)_1094	TG(26:1/16:0/18:0)+NH4	990.94	1094.18
TG	TG(26:1/16:0/18:1)_1024	TG(26:1/16:0/18:1)+NH4	988.92	1023.58
TG	TG(26:1/16:0/18:1)_1049	TG(26:1/16:0/18:1)+NH4	988.93	1049.38
TG	TG(26:1/16:0/18:1)_1063	TG(26:1/16:0/18:1)+NH4	988.93	1062.92
TG	TG(26:1/16:0/22:4)_1046	TG(26:1/16:0/22:4)+NH4	1038.94	1045.64
TG	TG(26:1/16:0/22:5)_1004	TG(26:1/16:0/22:5)+NH4	1036.93	1003.76
TG	TG(26:1/16:0/24:1)_1151	TG(26:1/16:0/24:1)+NH4	1073.02	1150.83
TG	TG(26:1/18:1/18:2)_1026	TG(26:1/18:1/18:2)+NH4	1012.93	1025.66
TG	TG(26:1/18:2/18:3)_968	TG(26:1/18:2/18:3)+NH4	1008.90	968.50
TG	TG(26:1/18:2/22:5)_965	TG(26:1/18:2/22:5)+NH4	1060.93	965.26
TG	TG(26:1/18:2/24:1)_1114	TG(26:1/18:2/24:1)+NH4	1097.02	1114.46
TG	TG(26:2/18:2/24:1)_1074	TG(26:2/18:2/24:1)+NH4	1095.01	1073.77
TG	TG(26:3/18:1/18:2)_944	TG(26:3/18:1/18:2)+NH4	1008.90	943.81
TG	TG(26:4/18:1/18:2)_933	TG(26:4/18:1/18:2)+NH4	1006.88	933.24
TG	TG(27:0/18:2/18:2)_1030	TG(27:0/18:2/18:2)+NH4	1026.94	1029.88
TG	TG(28:0)_403	TG(28:0)+NH4	544.46	403.01
TG	TG(28:1/18:1/24:0)_1178	TG(28:1/18:1/24:0)+NH4	1129.07	1178.45
TG	TG(28:1/18:2/24:0)_1178	TG(28:1/18:2/24:0)+NH4	1127.07	1177.51
TG	TG(30:0)_461	TG(30:0)+NH4	572.49	461.37
TG	TG(30:1)_407	TG(30:1)+NH4	570.47	406.80
TG	TG(32:2)_417	TG(32:2)+NH4	596.49	416.79
TG	TG(34:0)_420	TG(34:0)+H	611.52	419.59
TG	TG(34:2)_417	TG(34:2)+H	607.49	416.74
TG	TG(36:0e)_758	TG(36:0e)+NH4	642.60	757.56
TG	TG(38:0p)_752	TG(38:0p)+NH4	668.62	752.15
TG	TG(38:2)_650	TG(38:2)+K	701.51	649.85
TG	TG(38:3)_612	TG(38:3)+K	699.50	611.86
TG	TG(38:5)_378	TG(38:5)+NH4	674.54	378.32
TG	TG(39:3)_617	TG(39:3)+NH4	692.58	617.38
TG	TG(4:0/10:0/16:0)_534	TG(4:0/10:0/16:0)+NH4	572.49	533.53
TG	TG(4:0/12:0/16:0)_590	TG(4:0/12:0/16:0)+NH4	600.52	589.59

TG	TG(4:0/12:0/16:0)_590	TG(4:0/12:0/16:0)+Na	605.48	589.94
TG	TG(4:0/12:0/16:1)_462	TG(4:0/12:0/16:1)+NH4	598.50	462.33
TG	TG(4:0/12:0/18:2)_380	TG(4:0/12:0/18:2)+NH4	624.52	380.04
TG	TG(4:0/14:0/14:0)_369	TG(4:0/14:0/14:0)+H	583.49	368.86
TG	TG(4:0/14:0/14:1)_376	TG(4:0/14:0/14:1)+NH4	598.50	375.75
TG	TG(4:0/14:0/16:0)_645	TG(4:0/14:0/16:0)+Na	633.51	644.84
TG	TG(4:0/14:0/16:1)_589	TG(4:0/14:0/16:1)+NH4	626.53	589.05
TG	TG(4:0/14:0/18:2)_596	TG(4:0/14:0/18:2)+NH4	652.55	596.40
TG	TG(4:0/14:0/18:3)_555	TG(4:0/14:0/18:3)+NH4	650.54	555.16
TG	TG(4:0/14:1/14:1)_344	TG(4:0/14:1/14:1)+NH4	596.49	344.28
TG	TG(4:0/14:1/16:1)_539	TG(4:0/14:1/16:1)+NH4	624.52	539.03
TG	TG(4:0/14:1/18:2)_369	TG(4:0/14:1/18:2)+NH4	650.54	368.66
TG	TG(4:0/14:1/18:2)_389	TG(4:0/14:1/18:2)+NH4	650.54	389.34
TG	TG(4:0/14:1/18:3)_336	TG(4:0/14:1/18:3)+NH4	648.52	336.25
TG	TG(4:0/14:1/18:3)_371	TG(4:0/14:1/18:3)+NH4	648.52	371.34
TG	TG(4:0/15:0/16:0)_665	TG(4:0/15:0/16:0)+NH4	642.57	665.44
TG	TG(4:0/15:1/18:1)_472	TG(4:0/15:1/18:1)+K	687.50	471.94
TG	TG(4:0/16:0/16:0)_698	TG(4:0/16:0/16:0)+NH4	656.58	697.72
TG	TG(4:0/16:0/16:0)_698	TG(4:0/16:0/16:0)+Na	661.54	697.80
TG	TG(4:0/16:0/17:0)_715	TG(4:0/16:0/17:0)+NH4	670.60	715.42
TG	TG(4:0/16:0/17:1)_663	TG(4:0/16:0/17:1)+NH4	668.58	663.44
TG	TG(4:0/16:0/18:0)_737	TG(4:0/16:0/18:0)+NH4	684.61	737.02
TG	TG(4:0/16:0/18:2)_649	TG(4:0/16:0/18:2)+Na	685.54	649.18
TG	TG(4:0/16:0/18:3)_612	TG(4:0/16:0/18:3)+Na	683.52	611.66
TG	TG(4:0/16:0/18:3)_612	TG(4:0/16:0/18:3)+NH4	678.57	611.66
TG	TG(4:0/16:0/20:4)_635	TG(4:0/16:0/20:4)+NH4	704.58	635.33
TG	TG(4:0/16:1/17:1)_616	TG(4:0/16:1/17:1)+NH4	666.57	615.65
TG	TG(4:0/16:1/18:2)_596	TG(4:0/16:1/18:2)+K	699.50	596.40
TG	TG(4:0/16:1/18:2)_596	TG(4:0/16:1/18:2)+NH4	678.57	596.40
TG	TG(4:0/16:1/18:2)_596	TG(4:0/16:1/18:2)+Na	683.52	596.42
TG	TG(4:0/16:1/18:3)_556	TG(4:0/16:1/18:3)+NH4	676.55	556.07
TG	TG(4:0/16:1/20:4)_580	TG(4:0/16:1/20:4)+NH4	702.57	580.16
TG	TG(4:0/18:1/20:4)_635	TG(4:0/18:1/20:4)+NH4	730.60	634.59
TG	TG(4:0/18:1/22:5)_633	TG(4:0/18:1/22:5)+NH4	756.61	632.64
TG	TG(4:0/18:2/18:2)_604	TG(4:0/18:2/18:2)+K	725.51	603.72
TG	TG(4:0/18:2/18:2)_604	TG(4:0/18:2/18:2)+NH4	704.58	603.74
TG	TG(4:0/18:2/18:2)_604	TG(4:0/18:2/18:2)+Na	709.54	603.98
TG	TG(4:0/18:2/20:4)_587	TG(4:0/18:2/20:4)+NH4	728.58	587.42
TG	TG(40:0p)_790	TG(40:0p)+NH4	696.65	789.79
TG	TG(40:2p)_709	TG(40:2p)+NH4	692.62	708.51
TG	TG(41:4)_624	TG(41:4)+NH4	718.60	623.89
TG	TG(53:9)_717	TG(53:9)+H	859.68	717.47
TG	TG(54:0)_1034	TG(54:0)+K	929.79	1034.28
TG	TG(58:0)_1102	TG(58:0)+K	985.85	1101.59
TG	TG(58:4e)_1002	TG(58:4e)+NH4	942.89	1002.20
TG	TG(6:0/10:0/14:0)_336	TG(6:0/10:0/14:0)+NH4	572.49	336.18

TG	TG(6:0/10:0/14:0)_366	TG(6:0/10:0/14:0)+NH4	572.49	365.59
TG	TG(6:0/10:0/14:0)_366	TG(6:0/10:0/14:0)+Na	577.44	365.61
TG	TG(6:0/10:0/16:1)_416	TG(6:0/10:0/16:1)+NH4	598.50	415.88
TG	TG(6:0/10:0/18:1)_427	TG(6:0/10:0/18:1)+NH4	626.54	427.00
TG	TG(6:0/10:0/18:1)_445	TG(6:0/10:0/18:1)+NH4	626.54	444.77
TG	TG(6:0/10:0/18:2)_355	TG(6:0/10:0/18:2)+Na	629.48	354.68
TG	TG(6:0/10:0/18:2)_380	TG(6:0/10:0/18:2)+Na	629.48	380.07
TG	TG(6:0/10:0/18:2)_416	TG(6:0/10:0/18:2)+NH4	624.52	415.92
TG	TG(6:0/10:0/18:3)_356	TG(6:0/10:0/18:3)+NH4	622.50	355.69
TG	TG(6:0/10:0/18:3)_369	TG(6:0/10:0/18:3)+H	605.48	368.66
TG	TG(6:0/12:0/16:0)_420	TG(6:0/12:0/16:0)+NH4	628.55	420.33
TG	TG(6:0/12:0/16:0)_445	TG(6:0/12:0/16:0)+NH4	628.55	444.80
TG	TG(6:0/12:0/18:3)_420	TG(6:0/12:0/18:3)+H	633.51	420.32
TG	TG(6:0/14:0/16:0)_680	TG(6:0/14:0/16:0)+NH4	656.58	679.97
TG	TG(6:0/14:0/16:1)_625	TG(6:0/14:0/16:1)+NH4	654.57	625.41
TG	TG(6:0/14:0/16:1)_644	TG(6:0/14:0/16:1)+NH4	654.57	643.65
TG	TG(6:0/14:0/16:1)_644	TG(6:0/14:0/16:1)+Na	659.52	643.66
TG	TG(6:0/15:2/15:2)_372	TG(6:0/15:2/15:2)+H	631.49	372.10
TG	TG(6:0/15:2/15:2)_428	TG(6:0/15:2/15:2)+H	631.49	428.47
TG	TG(6:0/16:0/16:1)_694	TG(6:0/16:0/16:1)+Na	687.55	694.18
TG	TG(6:0/16:0/17:0)_759	TG(6:0/16:0/17:0)+NH4	698.63	758.90
TG	TG(6:0/16:0/17:1)_712	TG(6:0/16:0/17:1)+NH4	696.61	712.42
TG	TG(6:0/16:0/18:1)_733	TG(6:0/16:0/18:1)+NH4	710.63	733.46
TG	TG(6:0/16:0/18:2)_692	TG(6:0/16:0/18:2)+Na	713.57	692.31
TG	TG(6:0/16:0/18:2)_692	TG(6:0/16:0/18:2)+NH4	708.61	692.38
TG	TG(6:0/16:0/20:4)_677	TG(6:0/16:0/20:4)+NH4	732.61	677.33
TG	TG(6:0/16:0/22:5)_676	TG(6:0/16:0/22:5)+NH4	758.63	675.58
TG	TG(6:0/16:1/17:1)_670	TG(6:0/16:1/17:1)+NH4	694.60	670.06
TG	TG(6:0/16:1/18:2)_648	TG(6:0/16:1/18:2)+NH4	706.60	648.19
TG	TG(6:0/17:0/18:1)_750	TG(6:0/17:0/18:1)+NH4	724.65	750.19
TG	TG(6:0/17:1/18:1)_710	TG(6:0/17:1/18:1)+NH4	722.63	709.96
TG	TG(6:0/17:1/18:2)_668	TG(6:0/17:1/18:2)+NH4	720.61	667.62
TG	TG(6:0/18:2/18:2)_647	TG(6:0/18:2/18:2)+NH4	732.61	646.98
TG	TG(6:0/18:2/18:3)_608	TG(6:0/18:2/18:3)+NH4	730.60	608.08
TG	TG(62:2e)_1140	TG(62:2e)+NH4	1002.98	1140.20
TG	TG(8:0/18:2/18:2)_692	TG(8:0/18:2/18:2)+NH4	760.64	691.59
TG	TG(8:0/18:2/18:3)_655	TG(8:0/18:2/18:3)+NH4	758.63	654.64
TG	TG(9:0/9:0/18:2)_430	TG(9:0/9:0/18:2)+NH4	652.55	430.24
ZyE	ZyE(0:0)_273	ZyE(0:0)+H	385.35	272.95

Supplementary Table 2. Lipids identified by LC-MS in sow milk.

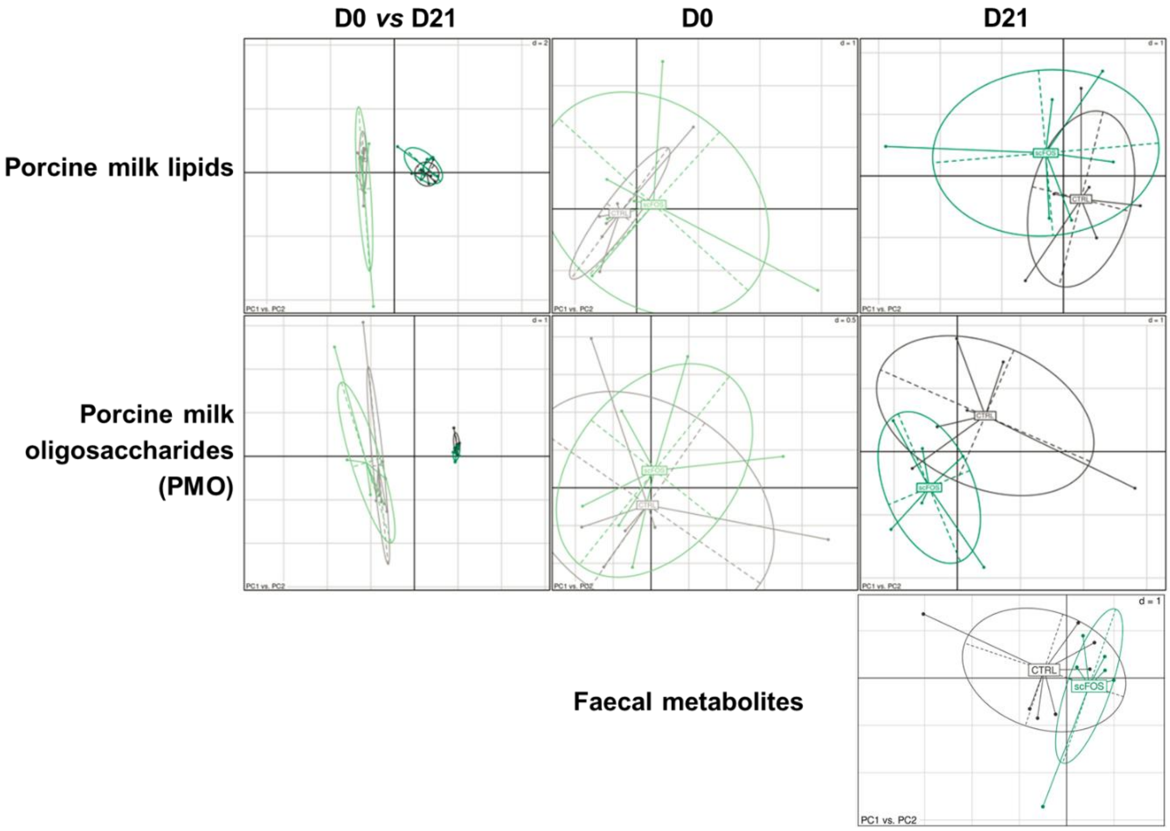

Supplementary Figure 1. Principal Component Analysis (PCA) done on variables related to porcine milk lipids and oligosaccharides at D0 and D21, and faecal metabolites at D21. CTRL: control group; scFOS: group supplemented with short chain fructo-oligosaccharides.

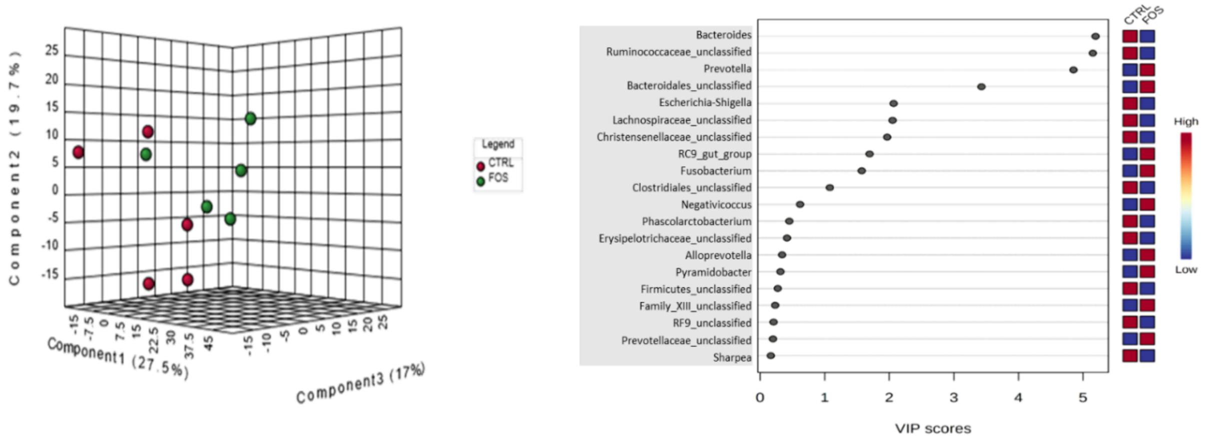

Supplementary Figure 2. Faecal microbiota composition (genus level) of D21 piglet. sPLS-DA analysis (left panel) and the 20 discriminant variables (right panel). CTRL: control group; scFOS: group supplemented with short chain fructo-oligosaccharides.

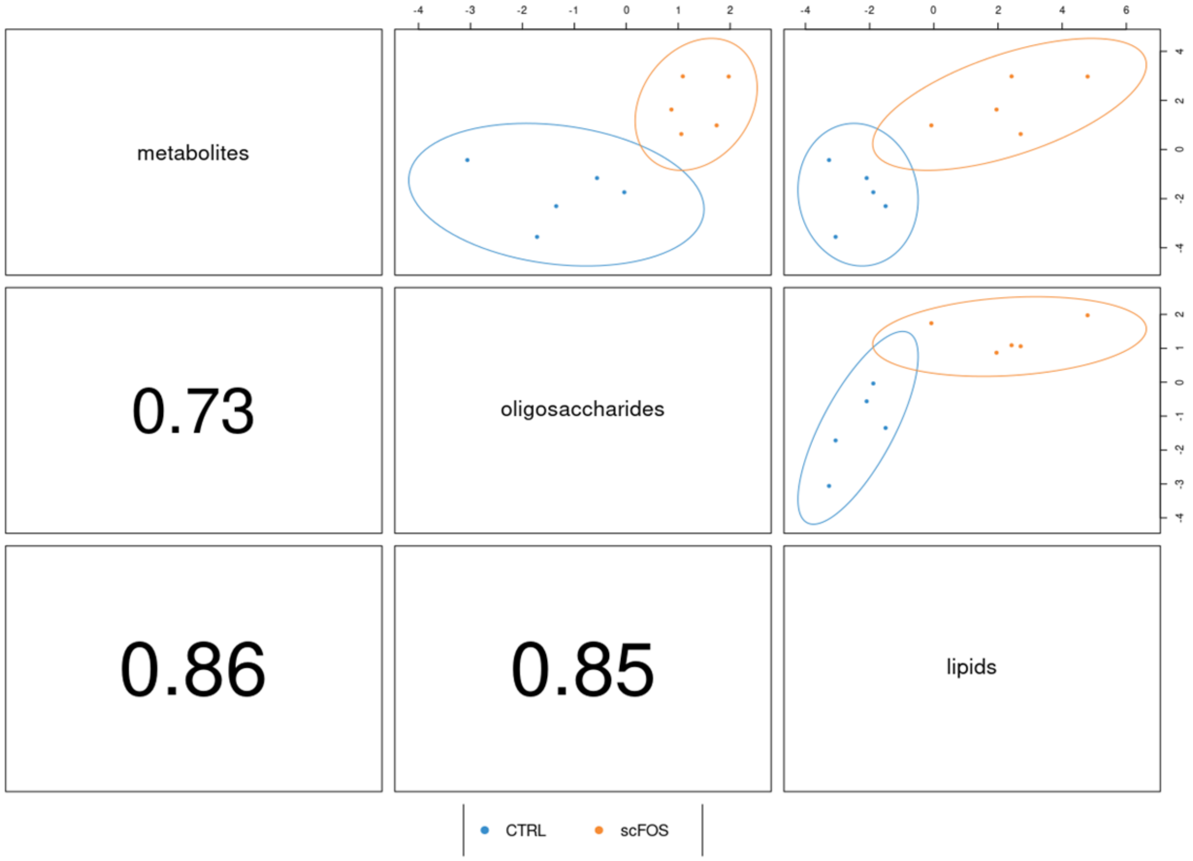

Supplementary Figure 3. Correlations between data blocks on the first component computed by sPLS-DA analysis from mixOmics R package. CTRL: control group; scFOS: group supplemented with short chain fructo-oligosaccharides. Oligosaccharide and lipid blocks from D21 sow milk and metabolite data block from piglet faeces. The lower triangular panel indicated the Pearson's correlation coefficient, the upper triangular panel is the scatter plot from values computed on the first component of sPLS-DA.

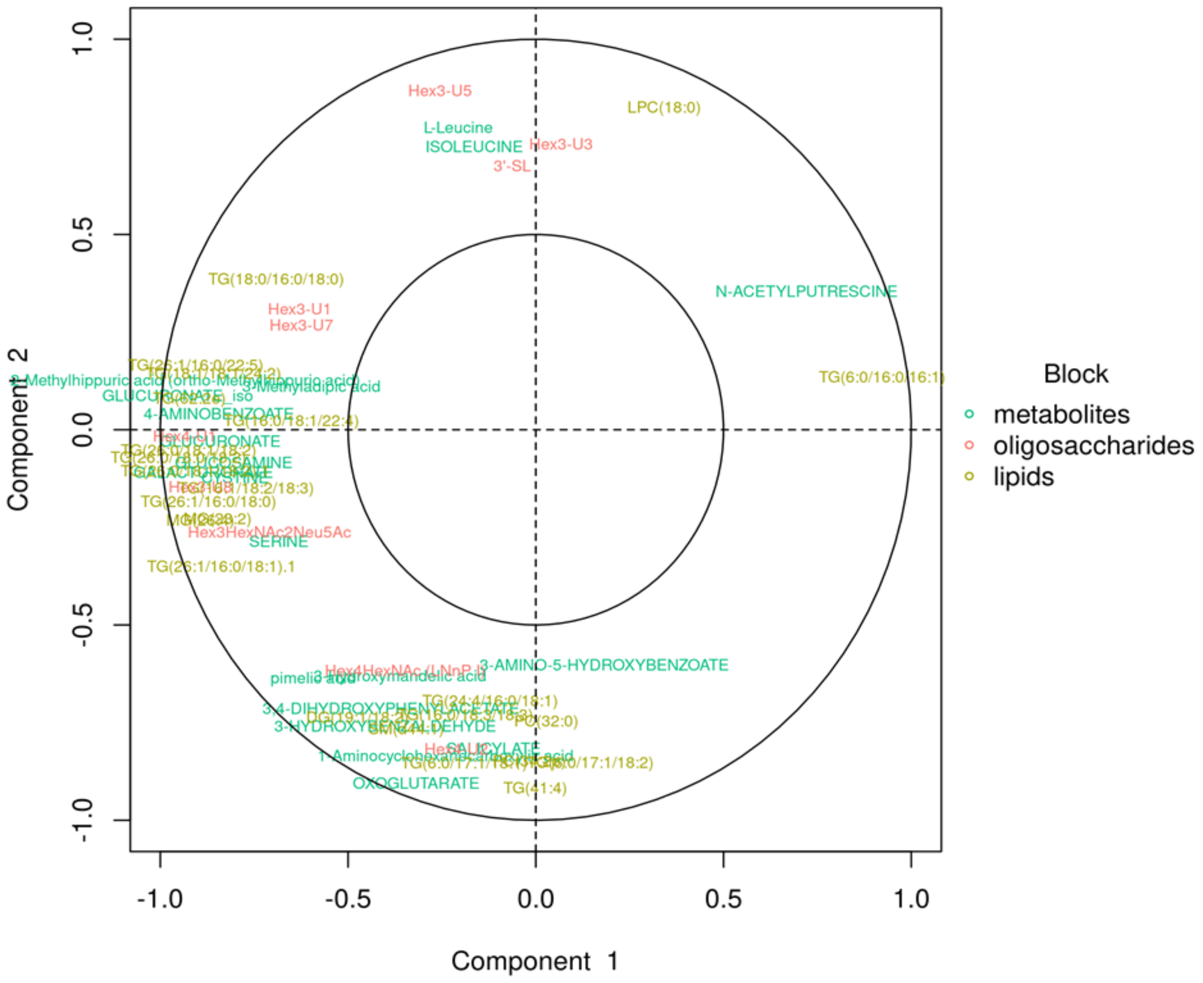

Supplementary Figure 4. Correlation circle plots of variables to the first two components computed by sPLS-DA analysis (detailed in Materials and Methods section).
